# Emergency and critical care services in Tanzania: a survey of ten hospitals

**DOI:** 10.1186/1472-6963-13-140

**Published:** 2013-04-16

**Authors:** Tim Baker, Edwin Lugazia, Jaran Eriksen, Victor Mwafongo, Lars Irestedt, David Konrad

**Affiliations:** 1 Department of Physiology and Pharmacology, Section for Anaesthesiology and Intensive Care Medicine, Karolinska Institute, Stockholm, Sweden; 2 Department of Anaesthesia, Intensive Care and Surgical Services, Karolinska University Hospital, Stockholm, 171 76, Sweden; 3 Department of Anaesthesia and Intensive Care, Muhimbili University of Health and Allied Sciences, Dar es Salaam, Tanzania; 4 Department of Public Health Sciences, Karolinska Institute, Stockholm, Sweden

**Keywords:** Emergency medicine, Critical care, Health services, Quality of health care, Developing countries, Africa, Tanzania, Triage

## Abstract

**Background:**

While there is a need for good quality care for patients with serious reversible disease in all countries in the world, Emergency and Critical Care tends to be one of the weakest parts of health systems in low-income countries. We assessed the structure and availability of resources for Emergency and Critical Care in Tanzania in order to identify the priorities for improving care in this neglected specialty.

**Methods:**

Ten hospitals in four regions of Tanzania were assessed using a structured data collection tool. Quality was evaluated with standards developed from the literature and expert opinion.

**Results:**

Important deficits were identified in infrastructure, routines and training. Only 30% of the hospitals had an emergency room for adult and paediatric patients. None of the seven district and regional hospitals had a triage area or intensive care unit for adults. Only 40% of the hospitals had formal systems for adult triage and in less than one third were critically ill patients seen by clinicians more than once daily. In 80% of the hospitals there were no staff trained in adult triage or critical care. In contrast, a majority of equipment and drugs necessary for emergency and critical care were available in the hospitals (median 90% and 100% respectively. The referral/private hospitals tended to have a greater overall availability of resources (median 89.7%) than district/regional hospitals (median 70.6).

**Conclusions:**

Many of the structures necessary for Emergency and Critical Care are lacking in hospitals in Tanzania. Particular weaknesses are infrastructure, routines and training, whereas the availability of drugs and equipment is generally good. Policies to improve hospital systems for the care of emergency and critically ill patients should be prioritised.

## Background

Emergency and Critical Care (EaCC) is one of the weakest parts of health systems in low-income countries [1]. Defined as the care given in hospital to patients with serious reversible disease, EaCC encompasses both the emergency care on arrival plus the care of inpatients when in a critical state. There is a need for EaCC in all countries in the world, irrespective of local resources [2]. The global burden of critical illness is difficult to quantify but is especially high in developing countries [3,4]. Ninety percent of trauma deaths and ninety percent of deaths from pneumonia, meningitis and other infections occur in low or middle income countries [5,6]. Ninety-nine percent of global maternal mortality is in developing countries [7]. Seventy-Five percent of the 7.6 million children under 5 who die each year worldwide are in Africa or Asia [8]. Fifty percent of child deaths in hospitals in developing countries occur within 24 hours of arriving at the hospital [9].

Although the majority of critically ill patients in developing countries are children and young adults, reported fatality rates are high: mortality for severe head injury in Benin is 70% and mortality for septic shock in Tunisia is 82% [10]. Several studies have reported deficiencies in care for seriously ill children [11,12] and in emergency obstetric care [13] in low-income countries. However, to our knowledge, there are no published studies looking at the quality of EaCC across all disciplines and no single-country analyses of EaCC services [14].

Tanzania is a politically stable, low-income country in East Africa. With a population of 44 million, Tanzania is ranked 152 out of 187 countries in the Human Development Index [15] (see Table 1). While Tanzania has some discipline-specific guidelines such as for obstetrics [16] or malaria [17], there are no national guidelines specifically for EaCC. The state of the services provided for the critically ill in Tanzania is largely unknown. In this study we aim to describe the structure and availability of hospital resources for EaCC in Tanzania as a starting point for the identification of priorities for improving care for this neglected patient group.

**Table 1:** Tanzania demographics

**Population**	**44 million**
Gross National Income per capita (US Dollars)	1430
Life Expectancy at birth (years)	55
Physicians (per 100,000 population)	0.1
Under-five mortality rate (per 1000)	76
Maternal mortality ratio (per 100,000 live births)	460

All data from World Health Statistics 2012 [18].

## Methods

There are 21 regions in mainland Tanzania and 223 hospitals [19]. Ten hospitals were selected for study feasibility from four regions of the country using convenience sampling, ensuring a mix of district, regional, referral-level and private hospitals. The hospitals were studied on single day visits by two of the investigators (EL and TB) between October and November 2009 after prior telephone contact. The investigators conducted the first five visits together so as to harmonise the conduct of the visits and how to collect the data. The subsequent five visits were done by a single investigator (two by EL, three by TB). The data was collected using a structured tool which further ensured consistency in data collection. A structured interview was conducted with the head of the hospital and all areas of the hospital involved in EaCC were visited and the facilities assessed using a data collection tool divided into hospital specialties to ensure that the facilities for all patient groups were observed (available in the Additional file 1).

As there have not been any published Tanzanian or international guidelines specifically for EaCC in low-income countries, we first developed a set of standards. Three of the researchers (TB, DK, JE) reviewed the literature and extracted relevant standards from international guidelines for trauma [20], paediatrics [21,22], surgery [23,24] and anaesthesia [25]. We used an “expert group” process to revise these standards. The expert group was comprised of the six researchers plus ten anaesthesiologists and physicians currently working or with recent experience of working in low-income countries (see acknowledgements). The researchers conducted several face-to-face meetings and the expert group were consulted via email and telephone. Due to resource and time constraints we were unable to organise a formal Delphi method or a face-to-face meeting of the whole expert group. A final document of 104 indicators were decided upon that describe the minimal structural requirements for EaCC in low-income countries (available in the Additional file 2).

For the purposes of this study, 19 of the indicators were disregarded. This decision was taken by the researchers prior to data collection due to the risk that the many drug and equipment indicators (60 of the 104 indicators) would weigh the study too greatly towards the availability of these items. To assess whether the hospitals had extra resources beyond the minimum required for EaCC we selected a further ten indicators for “Advanced EaCC” from international best-practice guidelines [26]. Processes and outcomes of EaCC were not evaluated.

The indicators were divided into eight sections: infrastructure; human resources; training; drugs; equipment; routines; guidelines; support services. Where data was missing from four or more hospitals the indicator was excluded (apart from indicators in the routines and guidelines sections where missing data was judged likely to be due to an absence in the hospital). The hospitals were stratified into two groups: district/regional and referral/private as we hypothesised that resources would differ between the groups. We calculated the proportion of hospitals where each resource was available. We calculated a “resource availability score” for each hospital, subdivided into indicator sections, as percentages of available resources with missing data excluded. Due to the small sample size, inferential statistics were not used.

Ethical clearance was granted by the National Institute for Medical Research in Tanzania (NIMR/HQ/R.8a/Vol.IX/846) and permission for the study was granted by The Tanzanian Commission for Science and Technology (Costech) and district authorities in each study district. All interviewees gave informed consent to participate and were informed that they could withdraw from the study at any time.

## Results

### Hospital characteristics

Of the ten hospitals included in the study, four were district hospitals (secondary care), three were regional hospitals (tertiary care), two were national referral hospitals (quaternary care) and one was a privately owned mission hospital (Table 2). The hospitals were located in four regions with a total population of 6.8 million (18% of the population of mainland Tanzania) [19]. All the hospitals divided patients up into specialty with separate admissions systems for adults and children. Obstetric patients were seen directly in the obstetric wards and were excluded from the analysis.

**Table 2:** Hospital characteristics

	**Hospitals**									
	**1**	**2**	**3**	**4**	**5**	**6**	**7**	**8**	**9**	**10**
Type	District	District	District	District	Regional	Regional	Regional	Referral	Private	Referral
Region	1	2	2	2	3	4	1	2	2	4
Number of beds	<200	200-400	200-400	200-400	>400	200-400	200-400	>400	<200	>400
Number of specialist doctors (excluding anaesthesiologists)	2	6	6	8	7	1	5	>50	18	25
Number of specialist anaesthesiologists	0	0	0	0	0	0	0	6	0	1
Number of non-specialist clinician anaesthetists	0	3	3	1	1	3	3	12	0	4
ICU	No	No	No	No	No	No	No	Yes	Yes	Yes
Number of operating theatres	1	n/a	2	2	5	2	1	11	n/a	9

Number of beds divided into three groups: <200; 200-400; >400.

ICU Intensive Care Unit, locally defined by the hospitals.

n/a not available.

### Quality of EaCC

**Figure 1:**
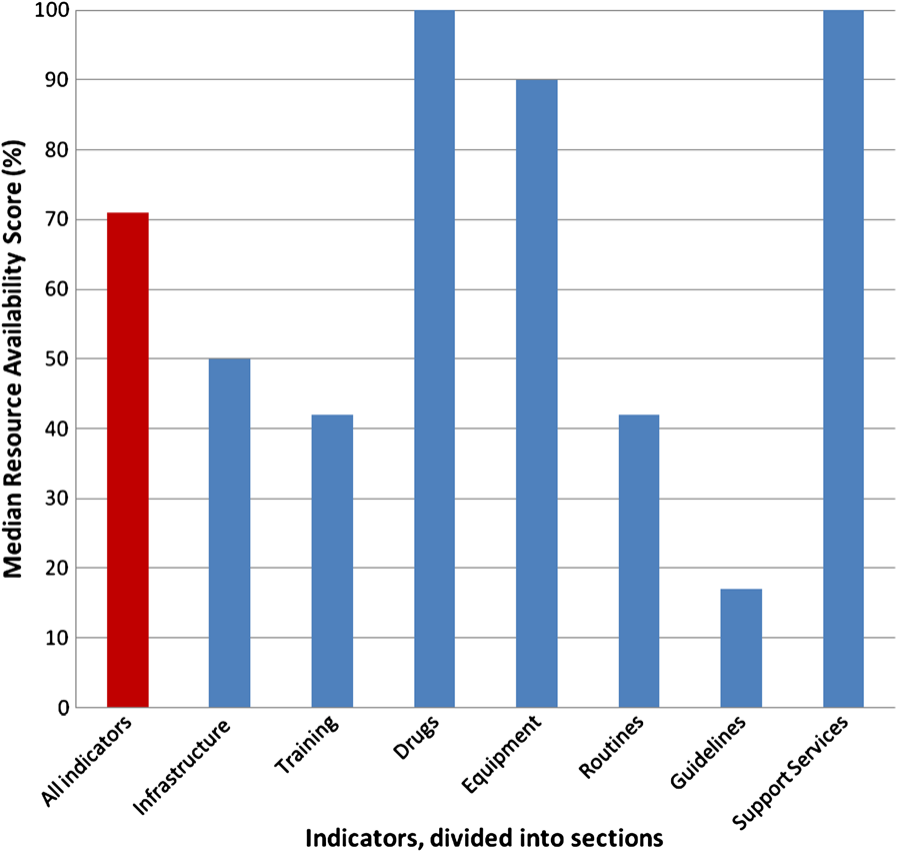
Emergency and critical care resource availability scores in ten hospitals in Tanzania.

Of the 104 indicators in the standards, 74 were used for the analysis. Nineteen equipment and drug indicators were disregarded to avoid weighting the study too greatly towards these items and eleven indicators were excluded due to missing data from four or more hospitals. The availability of each resource is shown in Table 3. A “Resource Availability Score” was calculated as the percentage of resources available in each hospital. The scores ranged from 57.1% to 92.1% with a median of 71.1% (Table 4). The referral/private hospitals had higher scores (median 89.7%) than district/regional hospitals (median 70.6%). Across all hospitals scores for infrastructure resources (50.0%) and for EaCC routines (42.2%) were lower than drugs (100%) and equipment (90.0%) (Figure 1).

**Table 3:** Percentage availability of resources for emergency and critical care

**Resource**			**All hospitals**	**District/regional hospitals**	**Referral/private hospitals**
			**n = 10**	**n = 7**	**n = 3**
			**%**	**%**	**%**
1	Infra-structure	Triage area for adults	30	0	100
2		Emergency/Resus Room or Area for adults	60	43	100
3		ICU or Area on a general ward for critically ill adults	50	29	100
4		Triage area for children	30*	43	100*
5		Emergency Room/Area for children	50	29	100
6		Paediatric ICU/area for critically ill children	90	86	100
7	Human Resources	Nurse always in the ER	100*	100*	100
8		Clinician always in the ER or on-call for ER	100*	100*	100
9		Designated Medical Head of ICU	-	-	100
10		There is a higher ratio of staff: patients on ICU than in any other ward	-	-	100
11	Training	Any staff trained in adult triage	20	29	0
12		Any staff formally trained in Emergency care of adults	40	29	67
13		Any staff formally trained in Critical Care for adults	20	0	67
14		Any staff trained in paediatric triage	40	43	33
15		Any staff formally trained in Emergency care of children	60	57	67
16		Any staff formally trained in Critical Care for children	80	71	100
17	Drugs	IV glucose	100*	100*	100
18		IV crystalloid	100*	100*	100
19		Diazepam	100*	100*	100
20		Paracetamol	100**	100**	100
21		IV penicillin	100	100	100
22		IV Gentamycin	100	100	100
23		IV Quinine	100**	100**	100
24		Adrenaline	100**	100**	100
25		Atropine	88*	83*	100
26		Salbutamol (for inhaler or nebuliser)	66*	50*	100
27		IV/IM opioid	100	100	100
28		Frusemide	100*	100*	100
29		Aminophylline	88*	83*	100
30		Insulin	70	57	100
31		Hydrocortisone	100	100	100
32	Equipment	Blood Pressure cuff	100	100	100
33		Stethoscope	100	100	100
34		IV cannulae	100	100	100
35		Bedside blood sugar strips/glucometer	70	71	67
36		Weighing scales	55*	57	50*
37		Suction	100*	100*	100
38		Gloves - clean	100	100	100
39		Thermometer	100*	100	100*
40		Urine Catheter	100	100	100
41		Oral Airway (Guedel)	60	43	100
42		Pulse oximeter	70	57	100
43		Naso-gastric Tubes	100	100	100
44		Oxygen concentrator/cylinder with face masks or nasal prongs and tubing	90	86	100
45		Electricity 24 hours/day	50	29	100
46		Running Water & soap	100	100	100
47	Routines	Formal system for categorising new adult patients according to urgency	40	14	100
48		Formal system for prioritising critically ill adults	44*	16*	100
49		Registration/Payment delayed until after triage & emergency treatment of critically ill adults	85***	80**	100*
50		Nurses do more frequent observations on critically ill adults	83****	66****	100
51		Ward rounds are done for critically ill adults at least twice a day	30	0	100
52		Formal system for categorising new paediatric patients according to urgency	57***	50*	100**
53		Formal system for prioritising critically ill children	-	50*	-
54		Registration/Payment delayed until after triage & emergency treatment of critically ill children	-	100****	-
55		Nurses do more frequent observations on critically ill children	77*	66*	100
56		Ward rounds are done for critically ill children at least twice a day	33*	0*	100
57		ICU admission criteria	-	-	50*
58		“Track and trigger” system on the wards for finding and referring critically ill patients to the ICU	-	-	0*
59	Guidelines	Triage Guidelines for adults	13**	0**	33
60		Guidelines for resuscitation/emergency care of adults	14***	0**	50*
61		Guidelines for managing seriously ill adults	20*****	0****	50*
62		Triage Guidelines for children	-	25***	-
63		Resuscitation Guidelines for children	-	25***	-
64		Guidelines for managing seriously ill children	-	33*	-
65		Guidelines for oxygen use	14***	25***	0
66	Support Services	Lab with facilities to measure Haemoglobin	100	100	100
67		Lab with facilities to measure Blood Glucose	100	100	100
68		Lab with facilities to measure Serum Urea/Creatinine, Sodium and Potassium	90	86	100
69		Chest X-ray facilities	100	100	100
70		System for emergency Blood Transfusion	100	100	100
71		System for making cross-matched blood available within one hour	80	86	67
72		System for testing donor blood for the viruses HIV, Hepatitis B & C	100***	100**	100*
73		Lab with facilities for microscopy & Bacterial Gram stain	100	100	100
74		Lab with facilities for bacterial culture and antibiotic sensitivities	80	71	100

Data given as a percentage of the hospitals where each resource was available.

* missing data is shown with one asterix (*) per missing hospital. Where data is missing for all hospitals in a section a dash is shown (-).

ER Emergency Room; ICU Intensive Care Unit; IV Intravenous; IM Intramuscular.

**Table 4:** Emergency and critical care resource availability scores*, by section

	**All hospitals**		**District/regional hospitals**	**Referral/private hospitals**
	**n = 10**		**n = 7**	**n = 3**
	**Median**	**Range**	**Median**	**Median**
**All indicators**	**71.1**	**57.1-92.1**	**70.6**	**89.7**
Infrastructure	**50.0**	16.7-100	50.0	100
Human Resources	**-**	-	-	100
Training	**41.7**	16.7-66.7	33.3	66,7
Drugs	**100**	71.4-100	86.7	100
Equipment	**90.0**	64.3-100	86.7	100
Routines	**42.2**	12.5-100	25.0	88.9
Guidelines	**17.1**	0-100	14.3	25
Support Services	**100**	75.0-100	100	100

* Resource availability scores calculated as a percentage of the resources available in each hospital. Averages calculated as the median score of the hospitals.

- missing data.

#### Infrastructure

In three hospitals there was a triage area for adults, and three hospitals had a triage area for children. Three hospitals had an emergency room (ER) for both children and adults. A further three had an ER for adults only and two hospitals had an ER for children only. Where an ER was not present the general outpatients department or the inpatient wards were used for emergency patients. Three hospitals had a ward that was designated as an Intensive Care Unit (ICU). The level of critical care provided on the ICUs varied: from ventilators and electronic patient monitors to a simple increase in the nurse: patient ratio and more frequent patient observations. In the other hospitals critically ill patients were cared for on general wards; two hospitals had a specific ward area for critically ill adults and nine hospitals had designated beds for critically ill children. The referral/private hospitals had a greater availability of infrastructure resources (median 100%) than district/regional (median 50%). None of the district hospitals had a triage area, Emergency Room or an ICU.

#### Human resources

Specialist Anaesthesiologists (the specialty in Tanzania responsible for Critical Care) were present in only the two referral hospitals. In two hospitals the anaesthetic service was staffed solely by nurses. In the other hospitals non-physician clinicians were present alongside nurses. These included Clinical Officers (staff with three years of college training) and Assistant Medical Officers (Clinical Officers with two further years of clinical training). Anaesthetic staffing was low in comparison to other medical staff (Table 2).

#### Training

In none of the hospitals had all the staff involved in EaCC been trained in triage, emergency care or critical care. The indicators were therefore adjusted to show if hospitals had “any” staff instead of “all” staff trained. Twenty percent of hospitals had at least one staff trained in adult triage and 20% had at least one staff trained in adult critical care. Training was most prevalent in Paediatric critical care with 80% of hospitals with at least one trained staff. Three hospitals (30%) had at least one staff who had been trained in the World Health Organisation’s paediatric training in “Emergency Triage & Treatment” (ETAT) [21].

#### Drugs and equipment

Intravenous fluids, parenteral opioids, diazepam, and antibiotics were available in all the hospitals where data was accessible as were blood pressure cuffs, naso-gastric tubes, suction machines and gloves. Oxygen was available in nine of the ten hospitals. Oro-pharyngeal airways were only found in six hospitals and salbutamol for inhalation in six out of nine hospitals. Reliable electricity with a backup generator was present in 50% of the hospitals (one of the district hospitals, one of the regional and all of the referral and private hospitals). The district/regional hospitals had slightly less availability of drugs and equipment than the referral/private hospitals (medians 86.7%, 100% respectively).

#### Routines and written guidelines

Forty percent of hospitals had formal systems of triage for adults and 44% had systems for prioritising the management of critically ill adults. Similar rates were seen for children. Registration and payment was delayed until after triage and emergency treatment at all hospitals for children and all except one for adults. Once admitted, 83% of hospitals had routines for increasing the frequency of observations for critically ill adults. However, ward rounds by clinicians were carried out more than once daily in only 30% and 33% of hospitals for critically ill adults and children respectively. There were no written guidelines for adult triage, emergency treatment or critical care in any of the district or regional hospitals.

#### Support services

All hospitals had the facilities for checking haemoglobin, conducting bacterial gram stain, and performing chest x-rays. 80% of hospitals could perform bacterial cultures and sensitivities, 90% could measure serum electrolytes. All hospitals could give emergency blood transfusions, 80% could do this within one hour of receiving a blood group specimen.

#### Advanced EaCC

Mechanical ventilators were present in 40% of hospitals, arterial blood gas monitoring in 20% and central venous pressure monitoring in 1 hospital of 10 (data not shown). A median of 25.0% of all indicators were available across all hospitals, with 20% in district/regional hospitals and 80% in referral/private hospitals.

## Discussion

We have shown that hospitals in Tanzania lack many structures necessary for Emergency and Critical Care (EaCC). Our study has found that the weakest parts of EaCC are an infrastructure that is not designed for managing critically ill patients, a lack of routines for the prioritisation and management of the critically ill and a low level of EaCC training among staff. In contrast, we have shown that hospitals are well stocked with drugs and equipment necessary for EaCC. Our study highlights the quality gap between the better resourced referral and private hospitals and the less resourced district and regional hospitals suggesting that while there is some capacity for EaCC in Tanzania, it hasn’t reached out to the smaller centres.

This study is, as far as we know, the first attempt to analyse the structural capacity for EaCC in a low-income country. As such its strengths are in highlighting this pressing need, to introduce standards for EaCC and to suggest likely causes of the reported weak performance and poor outcomes of EaCC in low-income countries [10,27]. Similar insights can be seen from the Tanzanian Service Provision Assessment Survey 2006 [28] where only 19% of hospitals nationwide had the facilities to support 24-hour emergency services and from studies focusing on paediatric inpatient care [12,29].

The sample size of ten hospitals is the study’s main weakness, limiting the generalisability to the whole country, and the single-country setting limits international generalisability. The experience of the authors is, however, that similar findings can be seen in many other hospitals in Tanzania. Recent studies in related disciplines have shown findings consistent with our results in the Gambia [30], Zambia [31], Mongolia [32] and in sub-Saharan Africa [33]. A further limitation is the lack of some data due to unavailability of information when the hospital visits were conducted. The processes and outcomes of EaCC were not observed in this study; the existence of certain equipment in a hospital does not mean it is in working order or in the correct place, used for the correct patients and at the correct time.

So why is the quality of EaCC so poor? It has not been prioritised politically or medically as it cuts across many disciplines, is not age or disease specific and there are very few Emergency or Intensive Care specialists. There is a belief that EaCC requires sophisticated or expensive equipment, although the basic EaCC that is the focus in this study such as adequate fluid resuscitation, intravenous dextrose for hypoglycaemia and oxygen for hypoxia are not expensive [34]. In our experience, fatalism among staff with low morale is common and self-fulfilling. Improving the service could lead to decreasing mortalities, which in turn could challenge fatalism. Vertical programs, while successfully tackling single diseases, have been less successful at strengthening health systems [35]. Programs for Essential Trauma Care [20], Emergency Obstetric Care [36], Hospital Care for Sick Children [22], Integrated Management for Adolescent and Adult Care [37] and Emergency and Essential Surgical Care [30,38] have all been initiated by the WHO in the past few years. A common theme is the view that improving care for very sick patients is urgently required and would have the potential of reducing preventable mortality. A focus on EaCC could be a way of coordinating efforts in these programs.

There is a potential for improvement in EaCC. Recognising very sick patients quickly and instituting simple treatments has been the focus of much recent work in high-income countries with the introduction of Rapid Response Teams [39,40]. The same principles apply in low-income settings: one study from Tanzania has shown that simple triage using vital signs can predict which patients have a high risk of mortality [41]. Reorganising a paediatric hospital admissions system in Malawi to include triage and emergency care reduced hospital mortality by 50% [42]. Intensive Care Units providing basic critical care are likely to be cost-effective due to a concentration of staff, skills and equipment close to the patients who need it most [43].

Improving levels of training is a vital issue in Tanzania and other low-income countries [44]. Critical Care is a subspecialty of Anaesthesia in Tanzania, and there are less than 20 specialist anaesthesiologists for a country of over 40 million people. Before 2009, when the first post-graduate training for doctors in Emergency Medicine was initiated, Tanzania had not trained any doctors in the speciality. Aside from the WHO’s paediatric ETAT program, there was little evidence of training courses in Tanzania in caring for emergency or critically ill patients. The almost complete lack of guidelines that we observed implies that standardised and evidenced based care is not being followed. Although guidelines alone have not always been seen to improve outcomes they may have the potential to improve quality of care if introduced through a multifaceted approach, targeting knowledge, motivation, resources, and the organisation of care [45-47]. These patients do not have the luxury of waiting, and introducing training, routines and guidelines to improve the processes of care may lead to reduced mortality and morbidity.

A striking finding in the study is how well stocked the hospitals were with drugs and equipment necessary for basic EaCC. This suggests that a lack of the basic tools for EaCC cannot be blamed for deficiencies in the quality of care. Drugs and equipment for advanced care were mostly absent, but this should be seen rather as a rational prioritisation of resources than as a deficiency.

## Conclusions

This study indicates to policy makers that improving the hospital systems for the care of emergency and critically ill patients in Tanzania should be prioritised. Improving the routines for managing these patients, increasing the human resources and training levels and improving the infrastructure may be the best ways to achieve this. Further research is required into the quality of the processes of EaCC and intervention studies are needed to assess the mortality and morbidity reductions and cost-effectiveness of improving EaCC.

## Abbreviations

EaCC: Emergency and critical care; ER: Emergency room; ETAT: Emergency triage and treatment; ICU: Intensive care unit.

## Competing interests

All the authors declare that they have no competing interests.

## Authors’ contributions

TB contributed to the conception and design of the study, acquired, analyzed and interpreted the data, and drafted and revised the manuscript. EL contributed to the design of the study, acquired the data and critically revised the manuscript. JE contributed to the conception and design of the study and critically revised the manuscript. VM contributed to the conception of the study and critically revised the manuscript. LI contributed to the conception of the study and critically revised the manuscript. DK contributed to the conception and design of the study, analyzed and interpreted the data and critically revised the manuscript. All authors read and approved the final manuscript.

## Pre-publication history

The pre-publication history for this paper can be accessed here:

http://www.biomedcentral.com/1472-6963/13/140/prepub

## Supplementary Material

Additional file 1 Data collection tool. Click here for file

Additional file 2 Structure standards for emergency and critical care in low income countries. Click here for file
