# Supplementary material for: Emergency and critical care services in Tanzania: a survey of ten hospitals
Source: BMC Health Serv Res. 2013 Apr 16;13:140. doi: 10.1186/1472-6963-13-140 (PMC3639070; doi:10.1186/1472-6963-13-140)
Supplement: Additional file 1 — Data collection tool. [file 1472-6963-13-140-S1.doc]

**The aim of this study is to investigate what facilities and processes there are in the hospital for managing patients who are emergencies or critically ill.**

**Our study includes care for all types of patients, irrespective of speciality or age. One section is specifically on the care for patients with severe sepsis.**

**We would like to talk to a few staff and see the facilities on each ward and in the OPDs/Emergency rooms.**

**All information is strictly confidential and the results will not indicate which hospitals were included**

**We would like to talk to the following people:**

**Section 1 Hospital Info Doctor in Charge**

**Section 2 Sepsis Doctor in Charge**

**Section 3 Medicine Head of Medicine**

**Section 4 Surgery/Trauma Head of Surgery**

**Section 5 Obstetrics Head of Obs**

**Section 6 Paediatrics Head of Paeds**

**Section 7 ICU Head of ICU**

**Section 8 Medical Records Head of Medical Records**

**Thank you for your cooperation!**

**Tim Baker**

**Edwin Lugazia**

**Investigator:** ....................................................  **Date:** .........................................................................

**Region:** ................................................................ **Hospital:** ..................................................................

**SECTION 1. THE HOSPITAL Interviewee: Grade:**

Type of hospital ⭘District ⭘Regional ⭘University ⭘Private ⭘Other (please write) ……………..

Number of beds ……………………… Population size in the catchment area ………………………

Admissions past 12 months ……………………… Outpatients seen past 12 months ………………………

Deliveries in past 12 months ………………………

Wards/beds

|  | Number of wards | Number of beds |
| --- | --- | --- |
| Paediatric |  |  |
| Surgical |  |  |
| Medical |  |  |
| Maternity |  |  |
| Gyn |  |  |
| ICU |  |  |
| Other |  |  |
| Other |  |  |
| TOTAL |  |  |
| Comments |  |  |

Operations

| How many operating theatres | …………………….. |
| --- | --- |
| Are the following operations carried out in the hospital: | |
| Caesarean section | ⭘yes ⭘no |
| Hysterectomy | ⭘yes ⭘no |
| Femur ORIF | ⭘yes ⭘no |
| Skull Burr holes | ⭘yes ⭘no |
| Splenectomy | ⭘yes ⭘no |
| Paediatric Circumcision | ⭘yes ⭘no |
| Paediatric Intestinal Obstruction | ⭘yes ⭘no |
| Comments |  |

**Number of Staff**

| Specialist Surgeons |  |
| --- | --- |
| Specialist Gynaecologists |  |
| Specialist Physicians |  |
| Specialist Paediatricians |  |
| Other specialists (which?) |  |
| MOs |  |
| Interns |  |
| AMOs |  |
| COs |  |
| Nurses - registered |  |
| Nurses - enrolled |  |
| Other staff |  |
| Comments |  |

Number of Anaesthetic Staff

| Specialist Anaesthesiologist |  |
| --- | --- |
| Trainee Anaesthesiologist |  |
| MO Anaesthesiologist |  |
| AMO Anaesthetist |  |
| CO Anaesthetist |  |
| Nurse Anaesthetist |  |
| Other Anaesthetist |  |
| Comments |  |

**Support Services**

Are there facilities and trained personnel to do:

| Hb | ⭘yes ⭘no |
| --- | --- |
| Glucose | ⭘yes ⭘no |
| Serum Urea/Creatinine, Sodium and Potassium | ⭘yes ⭘no |
| Chest X-ray | ⭘yes ⭘no |
| Direct microscopy | ⭘yes ⭘no |
| Gram stain | ⭘yes ⭘no |
| Bacterial culture and antibiotic sensitivities | ⭘yes ⭘no |
| **BLOOD** |  |
| Cross matching blood | ⭘yes ⭘no |
| Storing blood – a “blood bank” | ⭘yes ⭘no |
| Acquire units of blood urgently | ⭘yes ⭘no |
| How? |  |
| Can Emergency blood transfusions be given? | ⭘yes ⭘no |
| How long does it take for cross matched blood to be available from when the sample arrives in the lab? | ⭘ <1hr  ⭘ 1-2hrs  ⭘ >2hrs |
| Is Donor blood tested for HIV, Hep B & Hep C | ⭘yes ⭘no |
| Please describe how the blood is replaced in the blood bank or how a patient gets blood (eg from relative?) |  |
| Comments |  |

**ICU**

| Is there an ICU? | ⭘yes ⭘no |
| --- | --- |
| If yes – since when? |  |
| Which patients is it meant for? |  |
| If no – why not? |  |
| Comments |  |

**New patients**

When a new patient arrives, where do they go (mapokezi, then OPD, then ward?)

|  | Describe pathway of care |
| --- | --- |
| Medical |  |
| Surgical |  |
| Obstetric |  |
| Paediatric |  |
| Other |  |
| Comments |  |

When a **SERIOUSLY ILL** new patient arrives, where do they go (mapokezi, then OPD, then ward?)

|  | Describe pathway of care |
| --- | --- |
| Medical |  |
| Surgical |  |
| Obstetric |  |
| Paediatric |  |
| Other |  |
| Comments |  |

**OPINIONS – Triage, Emergency, ICU**

| Does the hospital have a triage system? | ⭘yes ⭘no |
| --- | --- |
| Details |  |
| How good do you think the hospital's triage is? | ⭘good ⭘average ⭘poor |
| What are the limiting factors to improving triage? (See below) |  |
| How important do you think it is to improve triage ? | ⭘very ⭘average ⭘not important |
| How good do you think the hospital's emergency care is? | ⭘good ⭘average ⭘poor |
| What are the limiting factors to improving emergency care? |  |
| How important do you think it is to improve emergency care? | ⭘very ⭘average ⭘not important |
| How good do you think the hospital's intensive care is? | ⭘good ⭘average ⭘poor |
| What are the limiting factors to improving intensive care? |  |
| How important do you think it is to improve intensive care? | ⭘very ⭘average ⭘not important |
| Comments  (eg poor organisation of care, excessive patient load, poor physical facilities, insufficient staff, lack of essential supplies and equipment, low staff morale, training, other) |  |

**OUTCOMES**

|  |  |
| --- | --- |
| In there past 12 months there have been: |  |
| How many admissions? |  |
| How many in-hospital deaths? |  |
| How many deaths within 24hrs of admission? |  |
| How many deaths were on the same day as admission? |  |
| How many "preventable deaths"? |  |
| How many admissions to the ICU? |  |
| Comments | ………………………………................. |

**SECTION 2 SEPSIS**

**Interviewee:**

**Grade:**

**Some of these questions are repeats, but it is needed to have them in same format as other global studies on sepsis care.**

Are the following drugs available in your hospital?

|  | Always | Sometimes | Never | Don’t know |
| --- | --- | --- | --- | --- |
| IV Ampicillin | ⭘ | ⭘ | ⭘ | ⭘ |
| IV Gentamycin | ⭘ | ⭘ | ⭘ | ⭘ |
| IV Chloramphenicol | ⭘ | ⭘ | ⭘ | ⭘ |
| IV Ceftriaxone, Cefotaxime or Ceftazidime | ⭘ | ⭘ | ⭘ | ⭘ |
| IV Piperacillin | ⭘ | ⭘ | ⭘ | ⭘ |
| IV Meropenem or other carbapenem | ⭘ | ⭘ | ⭘ | ⭘ |
| IV Hydrocortisone | ⭘ | ⭘ | ⭘ | ⭘ |
| Sodium Chloride, Ringers Lactate or other crystalloid | ⭘ | ⭘ | ⭘ | ⭘ |
| Gelatine, Dextran or other Colloid | ⭘ | ⭘ | ⭘ | ⭘ |
| Insulin | ⭘ | ⭘ | ⭘ | ⭘ |
| Oxygen | ⭘ | ⭘ | ⭘ | ⭘ |
| Blood Transfusion | ⭘ | ⭘ | ⭘ | ⭘ |
| Fresh Frozen Plasma | ⭘ | ⭘ | ⭘ | ⭘ |
| Platelets | ⭘ | ⭘ | ⭘ | ⭘ |
| Heparin or Low Molecular Weight Heparin | ⭘ | ⭘ | ⭘ | ⭘ |
| Ranitidine or other H2 receptor blocker | ⭘ | ⭘ | ⭘ | ⭘ |
| Omeprazole or other Proton Pump Inhibitor | ⭘ | ⭘ | ⭘ | ⭘ |
| IV morphine, pethidine or other IV opioid | ⭘ | ⭘ | ⭘ | ⭘ |
| Diazepam | ⭘ | ⭘ | ⭘ | ⭘ |
| Midazolam | ⭘ | ⭘ | ⭘ | ⭘ |
| Propofol | ⭘ | ⭘ | ⭘ | ⭘ |
| Thiopentone | ⭘ | ⭘ | ⭘ | ⭘ |
| Succinylcholine | ⭘ | ⭘ | ⭘ | ⭘ |
| Atracurium or other non-depolarising muscle relaxant | ⭘ | ⭘ | ⭘ | ⭘ |
| Noradrenaline | ⭘ | ⭘ | ⭘ | ⭘ |
| Dopamine | ⭘ | ⭘ | ⭘ | ⭘ |
| Dobutamine | ⭘ | ⭘ | ⭘ | ⭘ |
| Adrenaline | ⭘ | ⭘ | ⭘ | ⭘ |
| Vasopressin | ⭘ | ⭘ | ⭘ | ⭘ |
| Activated Protein C | ⭘ | ⭘ | ⭘ | ⭘ |

Can the following variables be monitored in your hospital?

|  | Always | Sometimes | Never | Don’t know |
| --- | --- | --- | --- | --- |
| Temperature | ⭘ | ⭘ | ⭘ | ⭘ |
| Non-invasive blood pressure | ⭘ | ⭘ | ⭘ | ⭘ |
| Invasive arterial blood pressure | ⭘ | ⭘ | ⭘ | ⭘ |
| Oxygen saturation | ⭘ | ⭘ | ⭘ | ⭘ |
| Central venous pressure | ⭘ | ⭘ | ⭘ | ⭘ |
| Cardiac output | ⭘ | ⭘ | ⭘ | ⭘ |
| Pulmonary arterial pressure | ⭘ | ⭘ | ⭘ | ⭘ |
| End tidal CO_2_ | ⭘ | ⭘ | ⭘ | ⭘ |

Can the following investigations be done in your hospital?

|  | Always | Sometimes | Never | Don’t know |
| --- | --- | --- | --- | --- |
| Blood slide for malaria parasites | ⭘ | ⭘ | ⭘ | ⭘ |
| Direct microscopy & gram stain | ⭘ | ⭘ | ⭘ | ⭘ |
| Bacteria culture | ⭘ | ⭘ | ⭘ | ⭘ |
| Antibiotic sensitivities | ⭘ | ⭘ | ⭘ | ⭘ |
| Blood glucose | ⭘ | ⭘ | ⭘ | ⭘ |
| Arterial blood gases | ⭘ | ⭘ | ⭘ | ⭘ |
| Blood lactate | ⭘ | ⭘ | ⭘ | ⭘ |
| Full blood count | ⭘ | ⭘ | ⭘ | ⭘ |
| Creatinine | ⭘ | ⭘ | ⭘ | ⭘ |
| Bilirubin | ⭘ | ⭘ | ⭘ | ⭘ |
| Prothrombin Time (INR) | ⭘ | ⭘ | ⭘ | ⭘ |
| Other coagulation test | ⭘ | ⭘ | ⭘ | ⭘ |

Is the following equipment available in your hospital?

|  | Always | Sometimes | Never | Don’t know |
| --- | --- | --- | --- | --- |
| X-ray | ⭘ | ⭘ | ⭘ | ⭘ |
| Ultrasound - Abdomen | ⭘ | ⭘ | ⭘ | ⭘ |
| Echocardiography | ⭘ | ⭘ | ⭘ | ⭘ |
| Mechanical Ventilator | ⭘ | ⭘ | ⭘ | ⭘ |
| Syringe pump | ⭘ | ⭘ | ⭘ | ⭘ |
| Fluid infuser | ⭘ | ⭘ | ⭘ | ⭘ |
| Peritoneal Dialysis | ⭘ | ⭘ | ⭘ | ⭘ |
| Hemodialysis/  hemofiltration | ⭘ | ⭘ | ⭘ | ⭘ |

Are the following items available in your hospital?

|  | Always | Sometimes | Never | Don’t know |
| --- | --- | --- | --- | --- |
| Venous cannula | ⭘ | ⭘ | ⭘ | ⭘ |
| IV fluid giving set | ⭘ | ⭘ | ⭘ | ⭘ |
| Urinary catheter | ⭘ | ⭘ | ⭘ | ⭘ |
| Gastric tube (NG-tube) | ⭘ | ⭘ | ⭘ | ⭘ |
| Endotracheal tube | ⭘ | ⭘ | ⭘ | ⭘ |
| Oxygen masks | ⭘ | ⭘ | ⭘ | ⭘ |
| Oxygen nasal cannula | ⭘ | ⭘ | ⭘ | ⭘ |
| Central venous catheter | ⭘ | ⭘ | ⭘ | ⭘ |
| Compression stockings | ⭘ | ⭘ | ⭘ | ⭘ |

**Thank you for your time. If possible, could we have a contact phone number in case we have more questions?**

**Name………………………………………………. Tel…………………………………………**

**Now we would like to talk to some other people – first Head of Medicine.**

**SECTION 3. MEDICINE**

**Interviewee: Grade:**

| **TRIAGE** |  |
| --- | --- |
| Where does a new patient go first, when they arrive? | ⭘mapokezi  ⭘nurse  ⭘clinician  ⭘other |
| What are the next steps - ie describe the care "pathway" for a new patient |  |
| If a "very sick" pat arrives, where do they go first? | ⭘mapokezi  ⭘nurse  ⭘clinician  ⭘other |
| Who decides a patient is "very sick"? | ⭘pat/relative  ⭘mapokezi  ⭘other |
| Is there a formal triage system? | ⭘yes ⭘no  If no go to *  If yes  go to ** |

* “NO“. TRIAGE IS INFORMAL

| Description of what happens if a „very sick patient“ arrives |  |
| --- | --- |
| Where is this done? |  |
| Are Airway, Breathing, Circulation, Conscious Level assessed | ⭘yes ⭘no |

**“YES“. THERE IS A FORMAL TRIAGE SYSTEM

| Where is the triage done? | ⭘triage area  ⭘mapokezi  ⭘OPD  ⭘ER  ⭘other |
| --- | --- |
| Description of the Triage area |  |
| What is the triage based on? |  |
| What are the categories of triage? |  |
| Who does the triage? | ⭘mapokezi  ⭘nurse  ⭘clinician  ⭘other |
| Are the staff trained in triage? | ⭘yes ⭘no |
| How, when, who? |  |
| Is triage done before registration? | ⭘yes ⭘no |
| Does triage assess Airway, Breathing, Circulation, Conscious Level | ⭘yes ⭘no |
| Are there Triage Guidelines? | ⭘yes ⭘no |
| Where are they kept? |  |

| **RESUSCITATION OF MEDICAL PATIENTS** | |
| --- | --- |
| If a "very sick" patient arrives or is found at triage, what is done? |  |
| Does the patient go first to registration? | ⭘yes ⭘no |
| Does the patient go to pay first? | ⭘yes ⭘no |
| Where are emergency or very sick patients first sent to? | ⭘Resus Room  ⭘nurse in OPD  ⭘Clinician in OPD  ⭘Ward  ⭘ICU  ⭘Other |
| If Resus Room or OPD Continue with below questions.  If OPD: change “Resus Room” to OPD.  If Ward or ICU skip below questions | |
| Describe how the Resus Room is used |  |
| If yes, where is the resus room located? | ⭘near entrance  ⭘away from entrance |
| Description of Resus Room |  |
| How many patients are seen in Resus room per day? |  |
| How is the resus room staffed with nurses? | ⭘nurse always in ER  ⭘nurse "on-call" to ER if emergency  ⭘nurse sometimes there |
| How is the resus room staffed with clinicians? | ⭘always in ER  ⭘"on-call" to ER if emergency  ⭘sometimes there |
| Which grade of clinicians? |  |
| How many nurses work at least sometimes in the resus room? |  |
| How many clinicians work at least sometimes in the resus room? |  |
| Are the Nurses trained in Resuscitation/Emergency Care? | ⭘yes ⭘no |
| If yes, give details - how many, when,what |  |
| Are the Clinicians trained in Resuscitation/Emergency Care? | ⭘yes ⭘no |
| If yes, give details - how many, when,what |  |
| If a "very sick" patient is identified, what is done? |  |
| Is a more senior clinician contacted? | ⭘yes ⭘no |
| If yes, who? |  |
| Does the patient pay for drugs & equipment first? | ⭘yes ⭘no |
| If the patient is unable to pay, does he get treated? | ⭘yes ⭘no |
| Is a "very sick" patient treated before others? | ⭘yes ⭘no |
| Can treatments be given in the resus room? | ⭘yes ⭘no |
| Are there guidelines for resuscitation/emergency care? | ⭘yes ⭘no |
| Where are the guidelines kept? | ⭘yes ⭘no |
| Where is a "very sick" patient sent to? | ⭘ ICU  ⭘ Ward  ⭘ other |

| **MEDICAL WARDS** |  |
| --- | --- |
| No. of wards |  |
| No. of beds |  |
| No. of nurses |  |
| No. of nurses in morning shift |  |
| No. of nurses in afternoon shift |  |
| No. of nurses in night shift? |  |
| No. of clinicians on ward & grades |  |
| Who does ward rounds? |  |
| How often are ward rounds done? |  |
| What are the 5 most common diagnoses on the ward? | 1.  2.  3.  4.  5. |
| What are the 5 most common causes of death on the ward? | 1.  2.  3.  4.  5. |
| How many deaths were there in the past 12 months? |  |
| No. of beds for seriously ill patients |  |
| Describe how these beds are different from other beds  (eg more rounds, more nurses, location, equipment etc) |  |
| How often do nurses do observations on the serious patients? |  |
| Which obs are done? |  |
| Is there an obs chart? | ⭘yes ⭘no |
| Is there a drugs chart? | ⭘yes ⭘no |
| Is there a fluid chart? | ⭘yes ⭘no |
| If a patient changes condition, who do the nurses call? |  |
| Do clinicians review patients at night / weekend? | ⭘yes ⭘no |
| Which grade of clinician? |  |
| Are the nurses allowed to begin treatment themselves? | ⭘yes ⭘no |
| If yes, which ones? |  |
| Are there guidelines for managing seriously ill patients? | ⭘yes ⭘no |
| For which conditions? |  |
| Where are they kept? |  |
| Is there senior backup for serious cases? | ⭘yes ⭘no |
| Who? |  |
| Can patients be admitted to ICU? How? Is there a "track and trigger" system on the wards for finding and referring patients to ICU? |  |
| Do patients pay before they use equipment eg cannula? | ⭘yes ⭘no |
| Do patients pay before get emergency drugs? | ⭘yes ⭘no |
| Are drugs available on the ward at night/w-end | ⭘yes ⭘no |
| Which ones? |  |
| Does the ward have Oxygen cylinders? | ⭘yes ⭘no |
| How many? |  |
| Does the ward have Oxygen concentrators? | ⭘yes ⭘no |
| How many? |  |
| Is there reliable electricity 24hrs/day? | ⭘yes ⭘no |
| Are there guidelines for oxygen use - ie which patients, how long is oxygen given etc? | ⭘yes ⭘no |

**DIRECT OBSERVATION MEDICAL OPD/ RESUS**

**Please can I see if the following drugs and equipment are in the Medical Resus Room / OPD today?**

| **Drugs** | yes | no |
| --- | --- | --- |
| ORS | ⭘ | ⭘ |
| IV glucose 5% | ⭘ | ⭘ |
| IV glucose 50% (or other concentration ≥ 10%) | ⭘ | ⭘ |
| IV crystalloid (Normal Saline Ringers Lactate) | ⭘ | ⭘ |
| Diazepam | ⭘ | ⭘ |
| Paracetamol | ⭘ | ⭘ |
| Salbutamol (for inhaler or nebuliser) | ⭘ | ⭘ |
| Parenteral Penicillin (or equivalent) | ⭘ | ⭘ |
| Parenteral Gentamycin (or equivalent) | ⭘ | ⭘ |
| Parenteral Quinine (or equivalent) | ⭘ | ⭘ |
| Other |  |  |

| **EQUIPMENT** | Yes | No |
| --- | --- | --- |
| Clock with secondhand |  |  |
| Gloves - clean |  |  |
| Gloves - sterile |  |  |
| Sharps disposal |  |  |
| Running water |  |  |
| Soap |  |  |
| Oral airway (Guedel) - adult size |  |  |
| Oral airway (Guedel) - paediatric size |  |  |
| Suction machine foot powered/electric |  |  |
| Suction tubing |  |  |
| Laryngoscope |  |  |
| Endotracheal Tubes - adult sizes |  |  |
| Endotracheal Tubes - paeds sizes |  |  |
| Rigid neck collar |  |  |
| Sandbags/Towel rolls and head restraints |  |  |
| Chest tube & underwater seal |  |  |
| Oxygen concentrator/cylinder |  |  |
| Oxygen masks, nasal prongs, tubing |  |  |
| Pulse oximeter |  |  |
| Bag valve mask (Ambu) |  |  |
| Stethoscope |  |  |
| Foetal stethoscope |  |  |
| BP cuff |  |  |
| IV cannulae,adult -eg 18G |  |  |
| IV cannulae,paeds -eg 22G, 24G |  |  |
| IV giving sets |  |  |
| Needles |  |  |
| Syringes - 2ml, 5ml |  |  |
| Urine catheters & bags |  |  |
| Gauze & bandages |  |  |
| Skin disinfectant |  |  |
| Torch |  |  |
| Electricity 24hrs/day |  |  |
| Telephone/other communication |  |  |
| Light suitable for clinical examination |  |  |
| Bedside blood sugar strips/glucometer |  |  |
| Weighing scales, adult |  |  |
| Weighing scales paeds |  |  |
| Thermometer |  |  |
| Triage Guidelines |  |  |
| Resuscitation Guidelines |  |  |
| Other guidelines |  |  |
| Other |  |  |

**DIRECT OBSERVATION MEDICAL WARD**

**Please can I see if the following drugs and equipment are on the Ward**

| **Drugs** | yes | no |
| --- | --- | --- |
| ORS | ⭘ | ⭘ |
| IV glucose 5% | ⭘ | ⭘ |
| IV glucose 50% (or other concentration ≥ 10%) | ⭘ | ⭘ |
| IV crystalloid (Normal Saline Ringers Lactate) | ⭘ | ⭘ |
| Diazepam | ⭘ | ⭘ |
| Paracetamol | ⭘ | ⭘ |
| Parenteral Penicillin (or equivalent) | ⭘ | ⭘ |
| Parenteral Gentamycin (or equivalent) | ⭘ | ⭘ |
| Parenteral Quinine (or equivalent) | ⭘ | ⭘ |
| Adrenaline | ⭘ | ⭘ |
| Atropine | ⭘ | ⭘ |
| Frusemide | ⭘ | ⭘ |
| Aminophylline | ⭘ | ⭘ |
| Salbutamol (for inhaler or nebuliser) | ⭘ | ⭘ |
| Hydrocortisone | ⭘ | ⭘ |
| Insulin | ⭘ | ⭘ |
| IV/IM opioids | ⭘ | ⭘ |
| Phenobarbital / Phenytoin | ⭘ | ⭘ |
| Other |  |  |

| **EQUIPMENT** | Yes | No |
| --- | --- | --- |
| Clock with secondhand | ⭘ | ⭘ |
| Gloves - clean | ⭘ | ⭘ |
| Gloves - sterile | ⭘ | ⭘ |
| Sharps disposal | ⭘ | ⭘ |
| Running water | ⭘ | ⭘ |
| Soap | ⭘ | ⭘ |
| Oral airway (Guedel) - adult size | ⭘ | ⭘ |
| Oral airway (Guedel) - paediatric size | ⭘ | ⭘ |
| Suction machine foot powered/electric | ⭘ | ⭘ |
| Suction tubing | ⭘ | ⭘ |
| Laryngoscope | ⭘ | ⭘ |
| Endotracheal Tubes - adult sizes | ⭘ | ⭘ |
| Endotracheal Tubes - paeds sizes | ⭘ | ⭘ |
| Rigid neck collar | ⭘ | ⭘ |
| Sandbags/Towel rolls and head restraints | ⭘ | ⭘ |
| Chest tube & underwater seal | ⭘ | ⭘ |
| Oxygen concentrator/cylinder | ⭘ | ⭘ |
| Oxygen masks, nasal prongs, tubing | ⭘ | ⭘ |
| Pulse oximeter | ⭘ | ⭘ |
| Bag valve mask (Ambu) | ⭘ | ⭘ |
| Stethoscope | ⭘ | ⭘ |
| Foetal stethoscope | ⭘ | ⭘ |
| BP cuff | ⭘ | ⭘ |
| IV cannulae,adult -eg 18G | ⭘ | ⭘ |
| IV cannulae,paeds -eg 22G, 24G | ⭘ | ⭘ |
| IV giving sets | ⭘ | ⭘ |
| Needles | ⭘ | ⭘ |
| Syringes - 2ml, 5ml | ⭘ | ⭘ |
| Urine catheters & bags | ⭘ | ⭘ |
| Gauze & bandages | ⭘ | ⭘ |
| Skin disinfectant | ⭘ | ⭘ |
| Torch | ⭘ | ⭘ |
| Electricity 24hrs/day | ⭘ | ⭘ |
| Telephone/other communication | ⭘ | ⭘ |
| Light suitable for clinical examination | ⭘ | ⭘ |
| Bedside blood sugar strips/glucometer | ⭘ | ⭘ |
| Weighing scales, adult | ⭘ | ⭘ |
| Weighing scales paeds | ⭘ | ⭘ |
| Thermometer | ⭘ | ⭘ |
| Guidelines for managing seriously ill patients | ⭘ | ⭘ |
| Obs, fluid, drugs charts | ⭘ | ⭘ |
| Other |  |  |

**SECTION 4. SURGERY/TRAUMA**

**Interviewee: Grade:**

| **TRIAGE** |  |
| --- | --- |
| Where does a new patient go first, when they arrive? | ⭘mapokezi  ⭘nurse  ⭘clinician  ⭘other |
| What are the next steps - ie describe the care "pathway" for a new patient |  |
| If a "very sick" pat arrives, where do they go first? | ⭘mapokezi  ⭘nurse  ⭘clinician  ⭘other |
| If a serious trauma victim arrives, where do they go first? |  |
| Who decides a patient is "very sick"? | ⭘pat/relative  ⭘mapokezi  ⭘other |
| Is there a formal triage system? | ⭘yes ⭘no  If no go to *  If yes  go to ** |

* “NO“. TRIAGE IS INFORMAL

| Description of what happens if a „very sick patient“ arrives |  |
| --- | --- |
| Where is this done? |  |
| Are Airway, Breathing, Circulation, Conscious Level assessed | ⭘yes ⭘no |

**“YES“. THERE IS A FORMAL TRIAGE SYSTEM

| Where is the triage done? | ⭘triage area  ⭘mapokezi  ⭘OPD  ⭘ER  ⭘other |
| --- | --- |
| Description of the Triage area |  |
| What is the triage based on? |  |
| What are the categories of triage? |  |
| Who does the triage? | ⭘mapokezi  ⭘nurse  ⭘clinician  ⭘other |
| Are the staff trained in triage? | ⭘yes ⭘no |
| How, when, who? |  |
| Is triage done before registration? | ⭘yes ⭘no |
| Does triage assess Airway, Breathing, Circulation, Conscious Level | ⭘yes ⭘no |
| Are there Triage Guidelines? | ⭘yes ⭘no |
| Where are they kept? |  |

| **RESUSCITATION OF SURGICAL PATIENTS** | |
| --- | --- |
| If a "very sick" patient arrives or is found at triage, what is done? |  |
| If a serious trauma patient arrives, what is done? |  |
| Does the patient go first to registration? | ⭘yes ⭘no |
| Does the patient go to pay first? | ⭘yes ⭘no |
| Where are emergency or very sick patients first sent to? | ⭘Resus Room  ⭘nurse in OPD  ⭘Clinician in OPD  ⭘Ward  ⭘ICU  ⭘Other |
| If Resus Room or OPD Continue with below questions.  If OPD: change “Resus Room” to OPD.  If Ward or ICU skip below questions | |
| Describe how the Resus Room is used |  |
| If yes, where is the resus room located? | ⭘near entrance  ⭘away from entrance |
| Description of Resus Room |  |
| How many patients are seen in Resus room per day? |  |
| How is the resus room staffed with nurses? | ⭘nurse always in ER  ⭘nurse "on-call" to ER if emergency  ⭘nurse sometimes there |
| How is the resus room staffed with clinicians? | ⭘always in ER  ⭘"on-call" to ER if emergency  ⭘sometimes there |
| Which grade of clinicians? |  |
| How many nurses work at least sometimes in the resus room? |  |
| How many clinicians work at least sometimes in the resus room? |  |
| Are the Nurses trained in Resuscitation/Emergency Care? | ⭘yes ⭘no |
| If yes, give details - how many, when,what |  |
| Are the Clinicians trained in Resuscitation/Emergency Care? | ⭘yes ⭘no |
| If yes, give details - how many, when,what |  |
| If a "very sick" patient is identified, what is done? |  |
| Is a more senior clinician contacted? | ⭘yes ⭘no |
| If yes, who? |  |
| Does the patient pay for drugs & equipment first? | ⭘yes ⭘no |
| If the patient is unable to pay, does he get treated? | ⭘yes ⭘no |
| Is a "very sick" patient treated before others? | ⭘yes ⭘no |
| Can treatments be given in the resus room? | ⭘yes ⭘no |
| Are there guidelines for resuscitation/emergency care? | ⭘yes ⭘no |
| Where are the guidelines kept? | ⭘yes ⭘no |
| Where is a "very sick" patient sent to? | ⭘ ICU  ⭘ Ward  ⭘ other |

| **SURGICAL WARDS** |  |
| --- | --- |
| No. of wards |  |
| No. of beds |  |
| No. of nurses |  |
| No. of nurses in morning shift |  |
| No. of nurses in afternoon shift |  |
| No. of nurses in night shift? |  |
| No. of clinicians on ward & grades |  |
| Who does ward rounds? |  |
| How often are ward rounds done? |  |
| What are the 5 most common diagnoses on the ward? | 1.  2.  3.  4.  5. |
| What are the 5 most common causes of death on the ward? | 1.  2.  3.  4.  5. |
| How many deaths have there been in the past 12 months? |  |
| No. of beds for seriously ill patients |  |
| Describe how these beds are different from other beds  (eg more rounds, more nurses, location, equipment etc) |  |
| How often do nurses do observations on the serious patients? |  |
| Which obs are done? |  |
| Is there an obs chart? | ⭘yes ⭘no |
| Is there a drugs chart? | ⭘yes ⭘no |
| Is there a fluid chart? | ⭘yes ⭘no |
| If a patient changes condition, who do the nurses call? |  |
| Do clinicians review patients at night / weekend? | ⭘yes ⭘no |
| Which grade of clinician? |  |
| Are the nurses allowed to begin treatment themselves? | ⭘yes ⭘no |
| If yes, which ones? |  |
| Are there guidelines for managing seriously ill patients? | ⭘yes ⭘no |
| For which conditions? |  |
| Where are they kept? |  |
| Is there senior backup for serious cases? | ⭘yes ⭘no |
| Who? |  |
| Can patients be admitted to ICU? How? Is there a "track and trigger" system on the wards for finding and referring patients to ICU? |  |
| Do patients pay before they use equipment eg cannula? | ⭘yes ⭘no |
| Do patients pay before get emergency drugs? | ⭘yes ⭘no |
| Are drugs available on the ward at night/w-end | ⭘yes ⭘no |
| Which ones? |  |
| Does the ward have Oxygen cylinders? | ⭘yes ⭘no |
| How many? |  |
| Does the ward have Oxygen concentrators? | ⭘yes ⭘no |
| How many? |  |
| Is there reliable electricity 24hrs/day? | ⭘yes ⭘no |
| Are there guidelines for oxygen use - ie which patients, how long is oxygen given etc? | ⭘yes ⭘no |

**DIRECT OBSERVATION SURGICAL OPD/ RESUS**

**Please can I see if the following drugs and equipment are in the Surgical Resus Room / OPD today?**

| **Drugs** | yes | no |
| --- | --- | --- |
| ORS | ⭘ | ⭘ |
| IV glucose 5% | ⭘ | ⭘ |
| IV glucose 50% (or other concentration ≥ 10%) | ⭘ | ⭘ |
| IV crystalloid (Normal Saline Ringers Lactate) | ⭘ | ⭘ |
| Diazepam | ⭘ | ⭘ |
| Paracetamol | ⭘ | ⭘ |
| Salbutamol (for inhaler or nebuliser) | ⭘ | ⭘ |
| Parenteral Penicillin (or equivalent) | ⭘ | ⭘ |
| Parenteral Gentamycin (or equivalent) | ⭘ | ⭘ |
| Parenteral Quinine (or equivalent) | ⭘ | ⭘ |
| Other |  |  |

| **EQUIPMENT** | Yes | No |
| --- | --- | --- |
| Clock with secondhand |  |  |
| Gloves - clean |  |  |
| Gloves - sterile |  |  |
| Sharps disposal |  |  |
| Running water |  |  |
| Soap |  |  |
| Oral airway (Guedel) - adult size |  |  |
| Oral airway (Guedel) - paediatric size |  |  |
| Suction machine foot powered/electric |  |  |
| Suction tubing |  |  |
| Laryngoscope |  |  |
| Endotracheal Tubes - adult sizes |  |  |
| Endotracheal Tubes - paeds sizes |  |  |
| Rigid neck collar |  |  |
| Sandbags/Towel rolls and head restraints |  |  |
| Chest tube & underwater seal |  |  |
| Oxygen concentrator/cylinder |  |  |
| Oxygen masks, nasal prongs, tubing |  |  |
| Pulse oximeter |  |  |
| Bag valve mask (Ambu) |  |  |
| Stethoscope |  |  |
| Foetal stethoscope |  |  |
| BP cuff |  |  |
| IV cannulae,adult -eg 18G |  |  |
| IV cannulae,paeds -eg 22G, 24G |  |  |
| IV giving sets |  |  |
| Needles |  |  |
| Syringes - 2ml, 5ml |  |  |
| Urine catheters & bags |  |  |
| Gauze & bandages |  |  |
| Skin disinfectant |  |  |
| Torch |  |  |
| Electricity 24hrs/day |  |  |
| Telephone/other communication |  |  |
| Light suitable for clinical examination |  |  |
| Bedside blood sugar strips/glucometer |  |  |
| Weighing scales, adult |  |  |
| Weighing scales paeds |  |  |
| Thermometer |  |  |
| Triage Guidelines |  |  |
| Resuscitation Guidelines |  |  |
| Other guidelines |  |  |
| Other |  |  |

**DIRECT OBSERVATION SURGICAL WARD**

**Please can I see if the following drugs and equipment are on the Ward**

| **Drugs** | yes | no |
| --- | --- | --- |
| ORS | ⭘ | ⭘ |
| IV glucose 5% | ⭘ | ⭘ |
| IV glucose 50% (or other concentration ≥ 10%) | ⭘ | ⭘ |
| IV crystalloid (Normal Saline Ringers Lactate) | ⭘ | ⭘ |
| Diazepam | ⭘ | ⭘ |
| Paracetamol | ⭘ | ⭘ |
| Parenteral Penicillin (or equivalent) | ⭘ | ⭘ |
| Parenteral Gentamycin (or equivalent) | ⭘ | ⭘ |
| Parenteral Quinine (or equivalent) | ⭘ | ⭘ |
| Adrenaline | ⭘ | ⭘ |
| Atropine | ⭘ | ⭘ |
| Frusemide | ⭘ | ⭘ |
| Aminophylline | ⭘ | ⭘ |
| Salbutamol (for inhaler or nebuliser) | ⭘ | ⭘ |
| Hydrocortisone | ⭘ | ⭘ |
| Insulin | ⭘ | ⭘ |
| IV/IM opioids | ⭘ | ⭘ |
| Phenobarbital / Phenytoin | ⭘ | ⭘ |
| Other |  |  |

| **EQUIPMENT** | Yes | No |
| --- | --- | --- |
| Clock with secondhand | ⭘ | ⭘ |
| Gloves - clean | ⭘ | ⭘ |
| Gloves - sterile | ⭘ | ⭘ |
| Sharps disposal | ⭘ | ⭘ |
| Running water | ⭘ | ⭘ |
| Soap | ⭘ | ⭘ |
| Oral airway (Guedel) - adult size | ⭘ | ⭘ |
| Oral airway (Guedel) - paediatric size | ⭘ | ⭘ |
| Suction machine foot powered/electric | ⭘ | ⭘ |
| Suction tubing | ⭘ | ⭘ |
| Laryngoscope | ⭘ | ⭘ |
| Endotracheal Tubes - adult sizes | ⭘ | ⭘ |
| Endotracheal Tubes - paeds sizes | ⭘ | ⭘ |
| Rigid neck collar | ⭘ | ⭘ |
| Sandbags/Towel rolls and head restraints | ⭘ | ⭘ |
| Chest tube & underwater seal | ⭘ | ⭘ |
| Oxygen concentrator/cylinder | ⭘ | ⭘ |
| Oxygen masks, nasal prongs, tubing | ⭘ | ⭘ |
| Pulse oximeter | ⭘ | ⭘ |
| Bag valve mask (Ambu) | ⭘ | ⭘ |
| Stethoscope | ⭘ | ⭘ |
| Foetal stethoscope | ⭘ | ⭘ |
| BP cuff | ⭘ | ⭘ |
| IV cannulae,adult -eg 18G | ⭘ | ⭘ |
| IV cannulae,paeds -eg 22G, 24G | ⭘ | ⭘ |
| IV giving sets | ⭘ | ⭘ |
| Needles | ⭘ | ⭘ |
| Syringes - 2ml, 5ml | ⭘ | ⭘ |
| Urine catheters & bags | ⭘ | ⭘ |
| Gauze & bandages | ⭘ | ⭘ |
| Skin disinfectant | ⭘ | ⭘ |
| Torch | ⭘ | ⭘ |
| Electricity 24hrs/day | ⭘ | ⭘ |
| Telephone/other communication | ⭘ | ⭘ |
| Light suitable for clinical examination | ⭘ | ⭘ |
| Bedside blood sugar strips/glucometer | ⭘ | ⭘ |
| Weighing scales, adult | ⭘ | ⭘ |
| Weighing scales paeds | ⭘ | ⭘ |
| Thermometer | ⭘ | ⭘ |
| Guidelines for managing seriously ill patients | ⭘ | ⭘ |
| Obs, fluid, drugs charts | ⭘ | ⭘ |
| Other |  |  |

**SECTION 5. OBSTETRICS**

**Interviewee: Grade:**

| How many deliveries were there in past 12 months? |  |
| --- | --- |
| How many Casaereans were done in the past 12 months? |  |
| How many mothers died in the past 12 months? |  |
|  |  |
| **TRIAGE** |  |
| Where does a new patient go first, when they arrive? | ⭘mapokezi  ⭘nurse  ⭘clinician  ⭘other |
| What are the next steps - ie describe the care "pathway" for a new patient |  |
| If a "very sick" pat arrives, where do they go first? | ⭘mapokezi  ⭘nurse  ⭘clinician  ⭘other |
| Who decides a patient is "very sick"? | ⭘pat/relative  ⭘mapokezi  ⭘other |
| Is there a formal triage system? | ⭘yes ⭘no  If no go to *  If yes  go to ** |

* “NO“. TRIAGE IS INFORMAL

| Description of what happens if a „very sick patient“ arrives |  |
| --- | --- |
| Where is this done? |  |
| Are Airway, Breathing, Circulation, Conscious Level assessed | ⭘yes ⭘no |

**“YES“. THERE IS A FORMAL TRIAGE SYSTEM

| Where is the triage done? | ⭘triage area  ⭘mapokezi  ⭘OPD  ⭘ER  ⭘other |
| --- | --- |
| Description of the Triage area |  |
| What is the triage based on? |  |
| What are the categories of triage? |  |
| Who does the triage? | ⭘mapokezi  ⭘nurse  ⭘clinician  ⭘other |
| Are the staff trained in triage? | ⭘yes ⭘no |
| How, when, who? |  |
| Is triage done before registration? | ⭘yes ⭘no |
| Does triage assess Airway, Breathing, Circulation, Conscious Level | ⭘yes ⭘no |
| Are there Triage Guidelines? | ⭘yes ⭘no |
| Where are they kept? |  |

| **RESUSCITATION OF OBSTETRIC PATIENTS** | |
| --- | --- |
| If a "very sick" patient arrives or is found at triage, what is done? |  |
| Does the patient go first to registration? | ⭘yes ⭘no |
| Does the patient go to pay first? | ⭘yes ⭘no |
| Where are emergency or very sick patients first sent to? | ⭘Resus Room  ⭘nurse in OPD  ⭘Clinician in OPD  ⭘Ward  ⭘ICU  ⭘Other |
| If Resus Room or OPD Continue with below questions.  If OPD: change “Resus Room” to OPD.  If Ward or ICU skip below questions | |
| Describe how the Resus Room is used |  |
| If yes, where is the resus room located? | ⭘near entrance  ⭘away from entrance |
| Description of Resus Room |  |
| How many patients are seen in Resus room per day? |  |
| How is the resus room staffed with nurses? | ⭘nurse always in ER  ⭘nurse "on-call" to ER if emergency  ⭘nurse sometimes there |
| How is the resus room staffed with clinicians? | ⭘always in ER  ⭘"on-call" to ER if emergency  ⭘sometimes there |
| Which grade of clinicians? |  |
| How many nurses work at least sometimes in the resus room? |  |
| How many clinicians work at least sometimes in the resus room? |  |
| Are the Nurses trained in Resuscitation/Emergency Care? | ⭘yes ⭘no |
| If yes, give details - how many, when,what |  |
| Are the Clinicians trained in Resuscitation/Emergency Care? | ⭘yes ⭘no |
| If yes, give details - how many, when,what |  |
| If a "very sick" patient is identified, what is done? |  |
| Is a more senior clinician contacted? | ⭘yes ⭘no |
| If yes, who? |  |
| Does the patient pay for drugs & equipment first? | ⭘yes ⭘no |
| If the patient is unable to pay, does he get treated? | ⭘yes ⭘no |
| Is a "very sick" patient treated before others? | ⭘yes ⭘no |
| Can treatments be given in the resus room? | ⭘yes ⭘no |
| Are there guidelines for resuscitation/emergency care? | ⭘yes ⭘no |
| Where are the guidelines kept? | ⭘yes ⭘no |
| Where is a "very sick" patient sent to? | ⭘ ICU  ⭘ Ward  ⭘ other |

| **OBSTETRIC WARD / MATERNITY** | |
| --- | --- |
| No. of wards |  |
| No. of beds |  |
| No. of nurses |  |
| No. of nurses in morning shift |  |
| No. of nurses in afternoon shift |  |
| No. of nurses in night shift? |  |
| No. of clinicians on ward & grades |  |
| Who does ward rounds? |  |
| How often are ward rounds done? |  |
| What are the 5 most common diagnoses on the ward? | 1.  2.  3.  4.  5. |
| What are the 5 most common causes of death on the ward? | 1.  2.  3.  4.  5. |
| No. of beds for seriously ill patients |  |
| Describe how these beds are different from other beds  (eg more rounds, more nurses, location, equipment etc) |  |
| How often do nurses do observations on the serious patients? |  |
| Which obs are done? |  |
| Is there an obs chart? | ⭘yes ⭘no |
| Is there a drugs chart? | ⭘yes ⭘no |
| Is there a fluid chart? | ⭘yes ⭘no |
| If a patient changes condition, who do the nurses call? |  |
| Do clinicians review patients at night / weekend? | ⭘yes ⭘no |
| Which grade of clinician? |  |
| Are the nurses allowed to begin treatment themselves? | ⭘yes ⭘no |
| If yes, which ones? |  |
| Are there guidelines for managing seriously ill patients? | ⭘yes ⭘no |
| For which conditions? |  |
| Where are they kept? |  |
| Is there senior backup for serious cases? | ⭘yes ⭘no |
| Who? |  |
| Can patients be admitted to ICU? How? Is there a "track and trigger" system on the wards for finding and referring patients to ICU? |  |
| Do patients pay before they use equipment eg cannula? | ⭘yes ⭘no |
| Do patients pay before get emergency drugs? | ⭘yes ⭘no |
| Are drugs available on the ward at night/w-end | ⭘yes ⭘no |
| Which ones? |  |
| Does the ward have Oxygen cylinders? | ⭘yes ⭘no |
| How many? |  |
| Does the ward have Oxygen concentrators? | ⭘yes ⭘no |
| How many? |  |
| Is there reliable electricity 24hrs/day? | ⭘yes ⭘no |
| Are there guidelines for oxygen use - ie which patients, how long is oxygen given etc? | ⭘yes ⭘no |
|  |  |
| Is there a room for premature babies? | ⭘yes ⭘no |
| Describe facilities |  |
| Is there a room for sick newborns? | ⭘yes ⭘no |
| Describe facilities |  |
| Are the guidelines for resuscitating newborns? | ⭘yes ⭘no |
| Where? |  |
| Is Oxygen used for resuscitating newborns? | ⭘yes ⭘no |
| Details |  |

**DIRECT OBSERVATION OBSTETRIC OPD/ RESUS**

**Please can I see if the following drugs and equipment are in the Resus Room / OPD today?**

| **Drugs** | yes | no |
| --- | --- | --- |
| ORS | ⭘ | ⭘ |
| IV glucose 5% | ⭘ | ⭘ |
| IV glucose 50% (or other concentration ≥ 10%) | ⭘ | ⭘ |
| IV crystalloid (Normal Saline Ringers Lactate) | ⭘ | ⭘ |
| Diazepam | ⭘ | ⭘ |
| Paracetamol | ⭘ | ⭘ |
| Salbutamol (for inhaler or nebuliser) | ⭘ | ⭘ |
| Parenteral Penicillin (or equivalent) | ⭘ | ⭘ |
| Parenteral Gentamycin (or equivalent) | ⭘ | ⭘ |
| Parenteral Quinine (or equivalent) | ⭘ | ⭘ |
| IV Magnesium | ⭘ | ⭘ |
| IV Oxytocin/Ergometrine | ⭘ | ⭘ |
| Other |  |  |

| **EQUIPMENT** | Yes | No |
| --- | --- | --- |
| Clock with secondhand |  |  |
| Gloves - clean |  |  |
| Gloves - sterile |  |  |
| Sharps disposal |  |  |
| Running water |  |  |
| Soap |  |  |
| Oral airway (Guedel) - adult size |  |  |
| Oral airway (Guedel) - paediatric size |  |  |
| Suction machine foot powered/electric |  |  |
| Suction tubing |  |  |
| Laryngoscope |  |  |
| Endotracheal Tubes - adult sizes |  |  |
| Endotracheal Tubes - paeds sizes |  |  |
| Rigid neck collar |  |  |
| Sandbags/Towel rolls and head restraints |  |  |
| Chest tube & underwater seal |  |  |
| Oxygen concentrator/cylinder |  |  |
| Oxygen masks, nasal prongs, tubing |  |  |
| Pulse oximeter |  |  |
| Bag valve mask (Ambu) |  |  |
| Stethoscope |  |  |
| Foetal stethoscope |  |  |
| BP cuff |  |  |
| IV cannulae,adult -eg 18G |  |  |
| IV cannulae,paeds -eg 22G, 24G |  |  |
| IV giving sets |  |  |
| Needles |  |  |
| Syringes - 2ml, 5ml |  |  |
| Urine catheters & bags |  |  |
| Gauze & bandages |  |  |
| Skin disinfectant |  |  |
| Torch |  |  |
| Electricity 24hrs/day |  |  |
| Telephone/other communication |  |  |
| Light suitable for clinical examination |  |  |
| Bedside blood sugar strips/glucometer |  |  |
| Weighing scales, adult |  |  |
| Weighing scales paeds |  |  |
| Thermometer |  |  |
| Triage Guidelines |  |  |
| Resuscitation Guidelines |  |  |
| Other guidelines |  |  |
| Other |  |  |

**DIRECT OBSERVATION OBSTETRIC WARD / MATERNITY**

**Please can I see if the following drugs and equipment are on the Ward**

| **Drugs** | yes | no |
| --- | --- | --- |
| ORS | ⭘ | ⭘ |
| IV glucose 5% | ⭘ | ⭘ |
| IV glucose 50% (or other concentration ≥ 10%) | ⭘ | ⭘ |
| IV crystalloid (Normal Saline Ringers Lactate) | ⭘ | ⭘ |
| Diazepam | ⭘ | ⭘ |
| Paracetamol | ⭘ | ⭘ |
| Parenteral Penicillin (or equivalent) | ⭘ | ⭘ |
| Parenteral Gentamycin (or equivalent) | ⭘ | ⭘ |
| Parenteral Quinine (or equivalent) | ⭘ | ⭘ |
| Adrenaline | ⭘ | ⭘ |
| Atropine | ⭘ | ⭘ |
| Frusemide | ⭘ | ⭘ |
| Aminophylline | ⭘ | ⭘ |
| Salbutamol (for inhaler or nebuliser) | ⭘ | ⭘ |
| Hydrocortisone | ⭘ | ⭘ |
| Insulin | ⭘ | ⭘ |
| IV/IM opioids | ⭘ | ⭘ |
| Phenobarbital / Phenytoin | ⭘ | ⭘ |
| Other |  |  |

| **EQUIPMENT** | Yes | No |
| --- | --- | --- |
| Clock with secondhand | ⭘ | ⭘ |
| Gloves - clean | ⭘ | ⭘ |
| Gloves - sterile | ⭘ | ⭘ |
| Sharps disposal | ⭘ | ⭘ |
| Running water | ⭘ | ⭘ |
| Soap | ⭘ | ⭘ |
| Oral airway (Guedel) - adult size | ⭘ | ⭘ |
| Oral airway (Guedel) - paediatric size | ⭘ | ⭘ |
| Suction machine foot powered/electric | ⭘ | ⭘ |
| Suction tubing | ⭘ | ⭘ |
| Laryngoscope | ⭘ | ⭘ |
| Endotracheal Tubes - adult sizes | ⭘ | ⭘ |
| Endotracheal Tubes - paeds sizes | ⭘ | ⭘ |
| Rigid neck collar | ⭘ | ⭘ |
| Sandbags/Towel rolls and head restraints | ⭘ | ⭘ |
| Chest tube & underwater seal | ⭘ | ⭘ |
| Oxygen concentrator/cylinder | ⭘ | ⭘ |
| Oxygen masks, nasal prongs, tubing | ⭘ | ⭘ |
| Pulse oximeter | ⭘ | ⭘ |
| Bag valve mask (Ambu) | ⭘ | ⭘ |
| Stethoscope | ⭘ | ⭘ |
| Foetal stethoscope | ⭘ | ⭘ |
| BP cuff | ⭘ | ⭘ |
| IV cannulae,adult -eg 18G | ⭘ | ⭘ |
| IV cannulae,paeds -eg 22G, 24G | ⭘ | ⭘ |
| IV giving sets | ⭘ | ⭘ |
| Needles | ⭘ | ⭘ |
| Syringes - 2ml, 5ml | ⭘ | ⭘ |
| Urine catheters & bags | ⭘ | ⭘ |
| Gauze & bandages | ⭘ | ⭘ |
| Skin disinfectant | ⭘ | ⭘ |
| Torch | ⭘ | ⭘ |
| Electricity 24hrs/day | ⭘ | ⭘ |
| Telephone/other communication | ⭘ | ⭘ |
| Light suitable for clinical examination | ⭘ | ⭘ |
| Bedside blood sugar strips/glucometer | ⭘ | ⭘ |
| Weighing scales, adult | ⭘ | ⭘ |
| Weighing scales paeds | ⭘ | ⭘ |
| Thermometer | ⭘ | ⭘ |
| Guidelines for managing seriously ill patients | ⭘ | ⭘ |
| Obs, fluid, drugs charts | ⭘ | ⭘ |
| Other |  |  |

**SECTION 6. PAEDIATRICS**

**Interviewee: Grade:**

| **TRIAGE** |  |
| --- | --- |
| Where does a new patient go first, when they arrive? | ⭘mapokezi  ⭘nurse  ⭘clinician  ⭘other |
| What are the next steps - ie describe the care "pathway" for a new patient |  |
| If a "very sick" pat arrives, where do they go first? | ⭘mapokezi  ⭘nurse  ⭘clinician  ⭘other |
| Who decides a patient is "very sick"? | ⭘pat/relative  ⭘mapokezi  ⭘other |
| Is there a formal triage system? | ⭘yes ⭘no  If no go to *  If yes  go to ** |

* “NO“. TRIAGE IS INFORMAL

| Description of what happens if a „very sick patient“ arrives |  |
| --- | --- |
| Where is this done? |  |
| Are Airway, Breathing, Circulation, Conscious Level assessed | ⭘yes ⭘no |

**“YES“. THERE IS A FORMAL TRIAGE SYSTEM

| Where is the triage done? | ⭘triage area  ⭘mapokezi  ⭘OPD  ⭘ER  ⭘other |
| --- | --- |
| Description of the Triage area |  |
| What is the triage based on? |  |
| What are the categories of triage? |  |
| Who does the triage? | ⭘mapokezi  ⭘nurse  ⭘clinician  ⭘other |
| Are the staff trained in triage? | ⭘yes ⭘no |
| How, when, who? |  |
| Is triage done before registration? | ⭘yes ⭘no |
| Does triage assess Airway, Breathing, Circulation, Conscious Level | ⭘yes ⭘no |
| Are there Triage Guidelines? | ⭘yes ⭘no |
| Where are they kept? |  |

| **RESUSCITATION OF PAEDIATRIC PATIENTS** | |
| --- | --- |
| If a "very sick" patient arrives or is found at triage, what is done? |  |
| Does the patient go first to registration? | ⭘yes ⭘no |
| Does the patient go to pay first? | ⭘yes ⭘no |
| Where are emergency or very sick patients first sent to? | ⭘Resus Room  ⭘nurse in OPD  ⭘Clinician in OPD  ⭘Ward  ⭘ICU  ⭘Other |
| If Resus Room or OPD Continue with below questions.  If OPD: change “Resus Room” to OPD.  If Ward or ICU skip below questions | |
| Describe how the Resus Room is used |  |
| If yes, where is the resus room located? | ⭘near entrance  ⭘away from entrance |
| Description of Resus Room |  |
| How many patients are seen in Resus room per day? |  |
| How is the resus room staffed with nurses? | ⭘nurse always in ER  ⭘nurse "on-call" to ER if emergency  ⭘nurse sometimes there |
| How is the resus room staffed with clinicians? | ⭘always in ER  ⭘"on-call" to ER if emergency  ⭘sometimes there |
| Which grade of clinicians? |  |
| How many nurses work at least sometimes in the resus room? |  |
| How many clinicians work at least sometimes in the resus room? |  |
| Are the Nurses trained in Resuscitation/Emergency Care? | ⭘yes ⭘no |
| If yes, give details - how many, when,what |  |
| Are the Clinicians trained in Resuscitation/Emergency Care? | ⭘yes ⭘no |
| If yes, give details - how many, when,what |  |
| If a "very sick" patient is identified, what is done? |  |
| Is a more senior clinician contacted? | ⭘yes ⭘no |
| If yes, who? |  |
| Does the patient pay for drugs & equipment first? | ⭘yes ⭘no |
| If the patient is unable to pay, does he get treated? | ⭘yes ⭘no |
| Is a "very sick" patient treated before others? | ⭘yes ⭘no |
| Can treatments be given in the resus room? | ⭘yes ⭘no |
| Are there guidelines for resuscitation/emergency care? | ⭘yes ⭘no |
| Where are the guidelines kept? | ⭘yes ⭘no |
| Where is a "very sick" patient sent to? | ⭘ ICU  ⭘ Ward  ⭘ other |

| **PAEDIATRIC WARDS** |  |
| --- | --- |
| No. of wards |  |
| No. of beds |  |
| No. of nurses |  |
| No. of nurses in morning shift |  |
| No. of nurses in afternoon shift |  |
| No. of nurses in night shift? |  |
| No. of clinicians on ward & grades |  |
| Who does ward rounds? |  |
| How often are ward rounds done? |  |
| What are the 5 most common diagnoses on the ward? | 1.  2.  3.  4.  5. |
| What are the 5 most common causes of death on the ward? | 1.  2.  3.  4.  5. |
| How many deaths have there been in the past 12 months? |  |
| No. of beds for seriously ill patients |  |
| Describe how these beds are different from other beds  (eg more rounds, more nurses, location, equipment etc) |  |
| How often do nurses do observations on the serious patients? |  |
| Which obs are done? |  |
| Is there an obs chart? | ⭘yes ⭘no |
| Is there a drugs chart? | ⭘yes ⭘no |
| Is there a fluid chart? | ⭘yes ⭘no |
| If a patient changes condition, who do the nurses call? |  |
| Do clinicians review patients at night / weekend? | ⭘yes ⭘no |
| Which grade of clinician? |  |
| Are the nurses allowed to begin treatment themselves? | ⭘yes ⭘no |
| If yes, which ones? |  |
| Are there guidelines for managing seriously ill patients? | ⭘yes ⭘no |
| For which conditions? |  |
| Where are they kept? |  |
| Is there senior backup for serious cases? | ⭘yes ⭘no |
| Who? |  |
| Can patients be admitted to ICU? How? Is there a "track and trigger" system on the wards for finding and referring patients to ICU? |  |
| Do patients pay before they use equipment eg cannula? | ⭘yes ⭘no |
| Do patients pay before get emergency drugs? | ⭘yes ⭘no |
| Are drugs available on the ward at night/w-end | ⭘yes ⭘no |
| Which ones? |  |
| Does the ward have Oxygen cylinders? | ⭘yes ⭘no |
| How many? |  |
| Does the ward have Oxygen concentrators? | ⭘yes ⭘no |
| How many? |  |
| Is there reliable electricity 24hrs/day? | ⭘yes ⭘no |
| Are there guidelines for oxygen use - ie which patients, how long is oxygen given etc? | ⭘yes ⭘no |

**DIRECT OBSERVATION PAEDIATRIC OPD/ RESUS**

**Please can I see if the following drugs and equipment are in the Paediatric Resus Room / OPD today?**

| **Drugs** | yes | no |
| --- | --- | --- |
| ORS | ⭘ | ⭘ |
| IV glucose 5% | ⭘ | ⭘ |
| IV glucose 50% (or other concentration ≥ 10%) | ⭘ | ⭘ |
| IV crystalloid (Normal Saline Ringers Lactate) | ⭘ | ⭘ |
| Diazepam | ⭘ | ⭘ |
| Paracetamol | ⭘ | ⭘ |
| Salbutamol (for inhaler or nebuliser) | ⭘ | ⭘ |
| Parenteral Penicillin (or equivalent) | ⭘ | ⭘ |
| Parenteral Gentamycin (or equivalent) | ⭘ | ⭘ |
| Parenteral Quinine (or equivalent) | ⭘ | ⭘ |
| Other |  |  |

| **EQUIPMENT** | Yes | No |
| --- | --- | --- |
| Clock with secondhand |  |  |
| Gloves - clean |  |  |
| Gloves - sterile |  |  |
| Sharps disposal |  |  |
| Running water |  |  |
| Soap |  |  |
| Oral airway (Guedel) - adult size |  |  |
| Oral airway (Guedel) - paediatric size |  |  |
| Suction machine foot powered/electric |  |  |
| Suction tubing |  |  |
| Laryngoscope |  |  |
| Endotracheal Tubes - adult sizes |  |  |
| Endotracheal Tubes - paeds sizes |  |  |
| Rigid neck collar |  |  |
| Sandbags/Towel rolls and head restraints |  |  |
| Chest tube & underwater seal |  |  |
| Oxygen concentrator/cylinder |  |  |
| Oxygen masks, nasal prongs, tubing |  |  |
| Pulse oximeter |  |  |
| Bag valve mask (Ambu) |  |  |
| Stethoscope |  |  |
| Foetal stethoscope |  |  |
| BP cuff |  |  |
| IV cannulae,adult -eg 18G |  |  |
| IV cannulae,paeds -eg 22G, 24G |  |  |
| IV giving sets |  |  |
| Needles |  |  |
| Syringes - 2ml, 5ml |  |  |
| Urine catheters & bags |  |  |
| Gauze & bandages |  |  |
| Skin disinfectant |  |  |
| Torch |  |  |
| Electricity 24hrs/day |  |  |
| Telephone/other communication |  |  |
| Light suitable for clinical examination |  |  |
| Bedside blood sugar strips/glucometer |  |  |
| Weighing scales, adult |  |  |
| Weighing scales paeds |  |  |
| Thermometer |  |  |
| Triage Guidelines |  |  |
| Resuscitation Guidelines |  |  |
| Other guidelines |  |  |
| Other |  |  |

**DIRECT OBSERVATION PAEDIATRIC WARD**

**Please can I see if the following drugs and equipment are on the Ward**

| **Drugs** | yes | no |
| --- | --- | --- |
| ORS | ⭘ | ⭘ |
| IV glucose 5% | ⭘ | ⭘ |
| IV glucose 50% (or other concentration ≥ 10%) | ⭘ | ⭘ |
| IV crystalloid (Normal Saline Ringers Lactate) | ⭘ | ⭘ |
| Diazepam | ⭘ | ⭘ |
| Paracetamol | ⭘ | ⭘ |
| Parenteral Penicillin (or equivalent) | ⭘ | ⭘ |
| Parenteral Gentamycin (or equivalent) | ⭘ | ⭘ |
| Parenteral Quinine (or equivalent) | ⭘ | ⭘ |
| Adrenaline | ⭘ | ⭘ |
| Atropine | ⭘ | ⭘ |
| Frusemide | ⭘ | ⭘ |
| Aminophylline | ⭘ | ⭘ |
| Salbutamol (for inhaler or nebuliser) | ⭘ | ⭘ |
| Hydrocortisone | ⭘ | ⭘ |
| Insulin | ⭘ | ⭘ |
| IV/IM opioids | ⭘ | ⭘ |
| Phenobarbital / Phenytoin | ⭘ | ⭘ |
| Other |  |  |

| **EQUIPMENT** | Yes | No |
| --- | --- | --- |
| Clock with secondhand | ⭘ | ⭘ |
| Gloves - clean | ⭘ | ⭘ |
| Gloves - sterile | ⭘ | ⭘ |
| Sharps disposal | ⭘ | ⭘ |
| Running water | ⭘ | ⭘ |
| Soap | ⭘ | ⭘ |
| Oral airway (Guedel) - adult size | ⭘ | ⭘ |
| Oral airway (Guedel) - paediatric size | ⭘ | ⭘ |
| Suction machine foot powered/electric | ⭘ | ⭘ |
| Suction tubing | ⭘ | ⭘ |
| Laryngoscope | ⭘ | ⭘ |
| Endotracheal Tubes - adult sizes | ⭘ | ⭘ |
| Endotracheal Tubes - paeds sizes | ⭘ | ⭘ |
| Rigid neck collar | ⭘ | ⭘ |
| Sandbags/Towel rolls and head restraints | ⭘ | ⭘ |
| Chest tube & underwater seal | ⭘ | ⭘ |
| Oxygen concentrator/cylinder | ⭘ | ⭘ |
| Oxygen masks, nasal prongs, tubing | ⭘ | ⭘ |
| Pulse oximeter | ⭘ | ⭘ |
| Bag valve mask (Ambu) | ⭘ | ⭘ |
| Stethoscope | ⭘ | ⭘ |
| Foetal stethoscope | ⭘ | ⭘ |
| BP cuff | ⭘ | ⭘ |
| IV cannulae,adult -eg 18G | ⭘ | ⭘ |
| IV cannulae,paeds -eg 22G, 24G | ⭘ | ⭘ |
| IV giving sets | ⭘ | ⭘ |
| Needles | ⭘ | ⭘ |
| Syringes - 2ml, 5ml | ⭘ | ⭘ |
| Urine catheters & bags | ⭘ | ⭘ |
| Gauze & bandages | ⭘ | ⭘ |
| Skin disinfectant | ⭘ | ⭘ |
| Torch | ⭘ | ⭘ |
| Electricity 24hrs/day | ⭘ | ⭘ |
| Telephone/other communication | ⭘ | ⭘ |
| Light suitable for clinical examination | ⭘ | ⭘ |
| Bedside blood sugar strips/glucometer | ⭘ | ⭘ |
| Weighing scales, adult | ⭘ | ⭘ |
| Weighing scales paeds | ⭘ | ⭘ |
| Thermometer | ⭘ | ⭘ |
| Guidelines for managing seriously ill patients | ⭘ | ⭘ |
| Obs, fluid, drugs charts | ⭘ | ⭘ |
| Other |  |  |

**SECTION 7. ICU**

| Does the hospital have an ICU? | ⭘yes ⭘no |
| --- | --- |
| **If no, skip this section** |  |
| If yes, since when? |  |
| How many beds are there on ICU? |  |
| Where is the ICU located?  (can tick several boxes) | ⭘ near ER/OPD  ⭘ near theatres  ⭘ near wards  ⭘ other |
| Description of ICU |  |
| Is there a medical head of ICU? | ⭘yes ⭘no |
| What is his/her grade & job title? |  |
| Does he/she have other duties than ICU? | ⭘yes ⭘no |
| If yes, what are they? (eg anaesthesia) |  |
| Has (s)he had formal training in Critical Care? | ⭘yes ⭘no |
| What training & when? |  |
| Are there any other clinicians stationed on the ICU? | ⭘yes ⭘no |
| Details. |  |
| No. of nurses in total on ICU |  |
| No. of nurses on average in morning shift? |  |
| No. of nurses on average in afternoon shift? |  |
| No. of nurses on average in the night shift? |  |
| How many nurses have formal training in Critical Care? |  |
| Details of training: |  |
| Do the nurses have duties other than ICU or rotate to other wards? |  |
| Does anyone else in the hospital have training in critical care? Details. |  |
| Who does ward rounds on the ICU? | ⭘ Head of ICU  ⭘ Physician Anaesthetist  ⭘ Other dr  ⭘ Non-physician Anaesthetist  ⭘ other |
| How often are ward rounds done? | ⭘ 1/day  ⭘ 2/day  ⭘ other |
| Is there always a named "on-call" clinician for ICU? | ⭘yes ⭘no |
| If a patient changes condition, who do the nurses call? | ⭘ on-call clinician  ⭘ pat's doctor  ⭘ head of icu  ⭘ other..................... |
| Is there senior backup for complex cases? | ⭘yes ⭘no |
| Do clinicians to review patients at night / weekend? | ⭘yes ⭘no |
| Who? | ⭘ on-call clinician  ⭘ pat's doctor  ⭘ head of icu  ⭘ other..................... |
| Are there ICU admission criteria? | ⭘yes ⭘no |
| What are they? |  |
| Where are they kept? |  |
| Are there ICU discharge criteria? | ⭘yes ⭘no |
| What are they? |  |
| Where are they kept? |  |
| Are Critically ill children cared for on the ICU? | ⭘yes ⭘no |
| Are Critically ill obstetric patients cared for on the ICU? | ⭘yes ⭘no |
| Are Critically ill pre-op surgical patients cared for on the ICU? | ⭘yes ⭘no |
| Are Critically ill post-op surgical patients cared for on the ICU? | ⭘yes ⭘no |
| Are Critically ill medical patients cared for on the ICU? | ⭘yes ⭘no |
| Are Critically ill trauma patients cared for on the ICU? | ⭘yes ⭘no |
| How are critically ill patients referred to ICU from the OPD/admissions? |  |
| How are critically ill patients referred to ICU from the wards? |  |
| Is there a "track and trigger" system on the wards for finding and referring critically ill patients to the ICU? | ⭘yes ⭘no |
| Are there guidelines for managing patients in ICU? | ⭘yes ⭘no |
| For which conditions? |  |
| Where are they kept? |  |
| Do patients have to pay before they use equipment eg cannula? | ⭘yes ⭘no |
| Is emergency equipment available at night weekends? | ⭘yes ⭘no |
| Which ones? |  |
| Does the ICU have a stock of emergency drugs? | ⭘yes ⭘no |
| Do patients have to pay before they receive emergency drugs? | ⭘yes ⭘no |
| Are emergency drugs available at night and weekends? | ⭘yes ⭘no |
| Does the ICU have Oxygen cylinders? | ⭘yes ⭘no |
| How many? |  |
| Where do they get filled? |  |
| How long does it take to get them filled? |  |
| What is the oxygen supply while the cylinders are away? |  |
| How often do they get filled? |  |
| Does the ICU have Oxygen concentrators? | ⭘yes ⭘no |
| How many? |  |
| How many are working reliably? |  |
| How do they get repaired? |  |
| How are spare parts purchased? |  |
| Is there reliable electricity 24hrs/day? | ⭘yes ⭘no |
| Are there guidelines for oxygen use - ie which patients receive oxygen? What are the criteria for giving oxygen? For how long is oxygen given to a single patient? | ⭘yes ⭘no |
| Is an observation chart is used for monitoring patients? | ⭘yes ⭘no |
| Is a drugs chart is used for recording treatment? | ⭘yes ⭘no |
| Is a fluid chart is used for monitoring fluid balance? | ⭘yes ⭘no |
| How often do nurses do observations? | ⭘ >1/hr  ⭘ 1/hr  ⭘ <1/hr  ⭘ other |
| Which obs are done? |  |
| Can nurses initiate treatments for ABCD themselves? | ⭘yes ⭘no |
| Which ones? |  |
| What is the routine if a patient has an airway problem? |  |
| What is the routine if a patient has hypoxia? |  |
| What is the routine if a patient is in shock? |  |
| What supportive care is given to patients? |  |
| What are routines for pain relief? |  |
| Does the hospital have regular audit of care for critically ill? | ⭘yes ⭘no |
| How regularly? |  |
| What is looked at? |  |
| Does the hospital have regular mortality meetings? | ⭘yes ⭘no |
| How regularly? |  |
| What is looked at? |  |
| How much does it cost a patient for 1 day on ICU? |  |
| How much does it cost the hospital to have a patient 1 day on ICU? |  |
| What are the 5 most common diagnoses on ICU? | 1.  2.  3.  4.  5. |
| What are the 5 most common causes of death on the ICU? | 1.  2.  3.  4.  5. |
| How many admissions to the ICU in the past 12 months? |  |
| How many deaths on the ICU in the past 12 months? |  |
| How many "readmissions" to ICU in the past 12 months? |  |
|  |  |

**DIRECT OBSERVATION ICU**

**Please can I see if the following drugs and equipment are on the ICU**

| **Drugs** | yes | no |
| --- | --- | --- |
| ORS | ⭘ | ⭘ |
| IV glucose 5% | ⭘ | ⭘ |
| IV glucose 50% (or other concentration ≥ 10%) | ⭘ | ⭘ |
| IV crystalloid (Normal Saline Ringers Lactate) | ⭘ | ⭘ |
| Diazepam | ⭘ | ⭘ |
| Paracetamol | ⭘ | ⭘ |
| Parenteral Penicillin (or equivalent) | ⭘ | ⭘ |
| Parenteral Gentamycin (or equivalent) | ⭘ | ⭘ |
| Parenteral Quinine (or equivalent) | ⭘ | ⭘ |
| Adrenaline | ⭘ | ⭘ |
| Atropine | ⭘ | ⭘ |
| Frusemide | ⭘ | ⭘ |
| Aminophylline | ⭘ | ⭘ |
| Salbutamol (for inhaler or nebuliser) | ⭘ | ⭘ |
| Hydrocortisone | ⭘ | ⭘ |
| Insulin | ⭘ | ⭘ |
| IV/IM opioids | ⭘ | ⭘ |
| Phenobarbital / Phenytoin | ⭘ | ⭘ |
| Ketamine | ⭘ | ⭘ |
| Lidocaine | ⭘ | ⭘ |
| Nifedipine or other anti-hypertensive | ⭘ | ⭘ |
| Naloxone | ⭘ | ⭘ |
| Thiopentone | ⭘ | ⭘ |
| Succinylcholine | ⭘ | ⭘ |
| Non-depolarising muscle relaxant | ⭘ | ⭘ |
| Oxytocin/Ergometrine | ⭘ | ⭘ |
| Magnesium Sulphate | ⭘ | ⭘ |
| Other |  |  |

| **EQUIPMENT** | Yes | No |
| --- | --- | --- |
| Clock with secondhand | ⭘ | ⭘ |
| Gloves - clean | ⭘ | ⭘ |
| Gloves - sterile | ⭘ | ⭘ |
| Sharps disposal | ⭘ | ⭘ |
| Running water | ⭘ | ⭘ |
| Soap | ⭘ | ⭘ |
| Oral airway (Guedel) - adult size | ⭘ | ⭘ |
| Oral airway (Guedel) - paediatric size | ⭘ | ⭘ |
| Suction machine foot powered/electric | ⭘ | ⭘ |
| Suction tubing | ⭘ | ⭘ |
| Laryngoscope | ⭘ | ⭘ |
| Endotracheal Tubes - adult sizes | ⭘ | ⭘ |
| Endotracheal Tubes - paeds sizes | ⭘ | ⭘ |
| Rigid neck collar | ⭘ | ⭘ |
| Sandbags/Towel rolls and head restraints | ⭘ | ⭘ |
| Chest tube & underwater seal | ⭘ | ⭘ |
| Oxygen concentrator/cylinder | ⭘ | ⭘ |
| Oxygen masks, nasal prongs, tubing | ⭘ | ⭘ |
| Pulse oximeter | ⭘ | ⭘ |
| Bag valve mask (Ambu) | ⭘ | ⭘ |
| Stethoscope | ⭘ | ⭘ |
| Foetal stethoscope | ⭘ | ⭘ |
| BP cuff | ⭘ | ⭘ |
| IV cannulae,adult -eg 18G | ⭘ | ⭘ |
| IV cannulae,paeds -eg 22G, 24G | ⭘ | ⭘ |
| IV giving sets | ⭘ | ⭘ |
| Needles | ⭘ | ⭘ |
| Syringes - 2ml, 5ml | ⭘ | ⭘ |
| Lumbar Puncture Needles | ⭘ | ⭘ |
| Urine catheters & bags | ⭘ | ⭘ |
| Gauze & bandages | ⭘ | ⭘ |
| Skin disinfectant | ⭘ | ⭘ |
| Torch | ⭘ | ⭘ |
| Electricity 24hrs/day | ⭘ | ⭘ |
| Telephone/other communication | ⭘ | ⭘ |
| Light suitable for clinical examination | ⭘ | ⭘ |
| Bedside blood sugar strips/glucometer | ⭘ | ⭘ |
| Weighing scales, adult | ⭘ | ⭘ |
| Weighing scales paeds | ⭘ | ⭘ |
| Refridgerator | ⭘ | ⭘ |
| Nasogastric Tubes | ⭘ | ⭘ |
| Thermometer | ⭘ | ⭘ |
| Guidelines for managing seriously ill patients | ⭘ | ⭘ |
| Observations Chart | ⭘ | ⭘ |
| Fluid Chart | ⭘ | ⭘ |
| Drugs charts | ⭘ | ⭘ |
| Other |  |  |

**SECTION 8. MEDICAL RECORDS**

|  | Medicine | Surgery | Obs | Paeds | Other | TOTAL |
| --- | --- | --- | --- | --- | --- | --- |
| In there past 12 months there have been: |  | | | | | |
| How many admissions? |  |  |  |  |  |  |
| How many OPD visits? |  |  |  |  |  |  |
| How many deliveries |  |  |  |  |  |  |
| How many in-hospital deaths? |  |  |  |  |  |  |
| How many deaths within 24hrs of admission? |  |  |  |  |  |  |
| How many deaths were on the same day as admission? |  |  |  |  |  |  |
| How many admissions to the ICU? |  |  |  |  |  |  |
| How many deaths on the ICU? |  |  |  |  |  |  |
| How many "readmissions" to ICU? |  |  |  |  |  |  |
| **Comments** |  |  |  |  |  |  |

**COMMENTS**
