# Supplementary material for: Emergency and critical care services in Tanzania: a survey of ten hospitals
Source: BMC Health Serv Res. 2013 Apr 16;13:140. doi: 10.1186/1472-6963-13-140 (PMC3639070; doi:10.1186/1472-6963-13-140)
Supplement: Additional file 2 — Structure standards for emergency and critical care in low income countries. [file 1472-6963-13-140-S2.doc]

**Structure Standards for Emergency and Critical Care in Low Income Countries**

| **The hospital should have** | | |  | |
| --- | --- | --- | --- | --- |
| **An infrastructure designed for managing emergency and critically ill adults and children** | | | |  |
| **Infrastructure**  **Indicators** | 1  2 | Designated Triage Area  For adults  For children | | |
|  | 3  4 | Designated Emergency Room / Area  For adults  For children | | |
|  | 5  6 | An ICU or a designated area of a ward for critically ill patients  For adults  For children | | |
| **Sufficient human resources for managing emergency and critically ill adults and children** | | | |  |
| **Human Resource**  **Indicators** | 7 | Nurse/other health worker either working in the ER or having the task of immediately going to the ER if a critically ill patient arrives | | |
|  | 8 | A clinician either working in the ER or being “on-call” for the ER if a critically ill patient arrives | | |
|  | 9 | A designated “Medical Head of ICU” | | |
|  | 10 | A higher ratio of staff: patients on ICU than on general wards | | |
| **Sufficiently trained staff for managing emergency and critically ill adults and children** | | | |  |
| **Training**  **Indicators** | 11 | Nurses and clinicians involved in adult triage have been trained in adult traige | | |
|  | 12 | Nurses and clinicians involved in emergency care of adults have undergone training in emergency care | | |
|  | 13 | Nurses and clinicians involved in critical care for adults have undergone training in critical care | | |
|  | 14 | Nurses and clinicians involved in paediatric triage have been trained in paediatric triage | | |
|  | 15 | Nurses and clinicians involved in emergency care of children have undergone training in emergency care | | |
|  | 16 | Nurses and clinicians involved in critical care for children have undergone training in paediatric critical care | | |
| **Essential drugs for emergency and critical care** | | | |  |
| **Drug**  **Indicators** | 17 | Oral Rehydration Solution | | |
|  | 18 | IV glucose | | |
|  | 19 | IV crystalloid (Normal Saline ± Ringers Lactate) | | |
|  | 20 | Diazepam | | |
|  | 21 | Paracetamol | | |
|  | 22 | Parenteral Penicillin (or equivalent) | | |
|  | 23 | Parenteral Gentamycin (or equivalent) | | |
|  | 24 | Parenteral Quinine (or other anti-malarial) | | |
|  | 25 | Ketamine | | |
|  | 26 | Lidocaine | | |
|  | 27 | Adrenaline | | |
|  | 28 | Atropine | | |
|  | 29 | Frusemide | | |
|  | 30 | Nifedipine or other anti-hypertensive | | |
|  | 31 | Aminophylline | | |
|  | 32 | Salbutamol (for inhaler or nebuliser) | | |
|  | 33 | Hydrocortisone | | |
|  | 34 | Insulin | | |
|  | 35 | IV/IM opioids | | |
|  | 36 | Naloxone | | |
|  | 37 | Thiopentone | | |
|  | 38 | Succinylcholine | | |
|  | 39 | Non-depolarising muscle relaxant | | |
|  | 40 | Oxytocin/Ergotamine | | |
|  | 41 | Magnesium Sulphate | | |
|  | 42 | Phenobarbital / Phenytoin | | |
| **Essential equipment for emergency and critical care** | | | |  |
| **Equipment**  **Indicators** | 43 | Clock with second hand | | |
|  | 44 | Gloves - clean | | |
|  | 45 | Gloves - sterile | | |
|  | 46 | Sharps disposal | | |
|  | 47 | Running water & soap | | |
|  | 48 | Oral airway (Guedel) – adult & paediatric sizes | | |
|  | 49 | Suction machine (foot powered or electric) & tubing | | |
|  | 50 | Laryngoscope (working) | | |
|  | 51 | Endotracheal Tubes – adult & paediatric sizes | | |
|  | 52 | Rigid neck collar or Sandbags/Towel rolls and head restraints | | |
|  | 53 | Chest tube & underwater seal (or equivalent) | | |
|  | 54 | Pulse oximeter | | |
|  | 55 | Bag valve mask (Ambu bag) | | |
|  | 56 | Stethoscope | | |
|  | 57 | Foetal stethoscope | | |
|  | 58 | Blood pressure cuff | | |
|  | 59 | IV cannulae – adult size (eg 18G) | | |
|  | 60 | IV cannulae – paediatric size (eg 22G, 24G) | | |
|  | 61 | IV giving sets | | |
|  | 62 | Needles | | |
|  | 63 | Syringes – 2ml & 5ml | | |
|  | 64 | Urine catheters & bags | | |
|  | 65 | Gauze & bandages | | |
|  | 66 | Skin disinfectant | | |
|  | 67 | Torch | | |
|  | 68 | Electricity 24hours/day | | |
|  | 69 | Light suitable for clinical examination | | |
|  | 70 | Bedside blood sugar testing device & strips | | |
|  | 71 | Weighing scales | | |
|  | 72 | Thermometer | | |
|  | 73 | Refrigerator | | |
|  | 74 | Nasogastric Tubes | | |
|  | 75 | Oxygen concentrator / cylinder with face masks or nasal prongs and tubing | | |
|  | 76 | System for ensuring continuous availability of oxygen (eg reserve electricity generator / reserve cylinders with good transport and refilling system) | | |
| **Routines for managing emergency and critically ill adults and children** | | | |  |
| **Routines**  **Indicators** | 77  78 | System for categorising patients according to clinical urgency (triage)  For adults  For children | | |
|  | 79  80 | System for prioritising the treatment of critically ill patients before stable patients  For adults  For children | | |
|  | 81  82 | Admission registration and payment delayed until after triage and emergency treatment  For adults  For children | | |
|  | 83 | ICU admission/discharge criteria | | |
|  | 84  85 | Nurses have a routine of frequent observations of the patients ( hourly or specified depending on clinical need)  For adults  For children | | |
|  | 86  87 | Clinicians check patients (ward rounds) at least twice a day  For adults  For children | | |
|  | 88 | There is a system for identifying critically ill patients on general wards and transferring to ICU (A “track and trigger” system) | | |
| **Guidelines for managing emergency and critically ill adults and children** | | | |  |
| **Guidelines**  **Indicators** | 89  90 | Guidelines for triage  For adults  For children | | |
|  | 91  92 | Guidelines for Emergency Care  For adults  For children | | |
|  | 93  94 | Guidelines for Critical Care  For adults  For children | | |
|  | 95 | Guidelines for Oxygen use | | |
| **Support Services for managing emergency and critically ill adults and children** | | | |  |
| **Support Services**  **Indicators** | 96 | Lab with facilities and trained personnel to measure Haemoglobin | | |
|  | 97 | Lab with facilities and trained personnel to measure blood glucose | | |
|  | 98 | Lab with facilities and personnel to measure Serum Urea/Creatinine, Sodium and Potassium | | |
|  | 99 | X-ray facilities and trained personnel for chest radiographs | | |
|  | 100 | System for emergency blood transfusion | | |
|  | 101 | System for making cross matched blood available within 1 hour of blood sample arriving in lab | | |
|  | 102 | System for testing donor blood for the viruses HIV, Hepatitis B & C | | |
|  | 103 | Lab with facilities and trained personnel to do direct microscopy & bacterial gram stain | | |
|  | 104 | Lab with facilities and trained personnel to do bacterial culture and antibiotic sensitivities | | |

IV intravenous; IM intramuscular;

| **Advanced Emergency & Critical Care Indicators**  **The hospital has:** | | |
| --- | --- | --- |
| 1 | Ventilator |  |
| 2 | Piperacillin/Meropenem |  |
| 3 | Colloid |  |
| 4 | Fresh Frozen Plasma |  |
| 5 | Propofol or Midazolam |  |
| 6 | Noradrenaline or Dobutamine |  |
| 7 | Invasive Blood Pressure Monitoring |  |
| 8 | Central Venous Pressure Monitoring |  |
| 9 | Aterial Blood Gas analysis |  |
| 10 | Syringe pump |  |

**References**
